# Effect of TAT-DOX-PEG irradiated gold nanoparticles conjugates on human osteosarcoma cells

**DOI:** 10.1038/s41598-020-63245-8

**Published:** 2020-04-20

**Authors:** Raoul V. Lupusoru, Daniela A. Pricop, Cristina M. Uritu, Adina Arvinte, Adina Coroaba, Irina Esanu, Mirela F. Zaltariov, Mihaela Silion, Cipriana Stefanescu, Mariana Pinteala

**Affiliations:** 1 Department of Pathophysiology, Faculty of Medicine, “Grigore T. Popa” University of Medicine and Pharmacy, 700115 Iasi, Romania; 2 Faculty of Physics, “Alexandru Ioan Cuza” University, 700506 Iasi, Romania; 3 Centre of Advanced Research in Bionanoconjugates and Biopolymers, “Petru Poni” Institute of Macromolecular Chemistry, 700487 Iasi, Romania; 4 Advanced Research and Development Center for Experimental Medicine (CEMEX), “Grigore T. Popa” University of Medicine and Pharmacy, 700115 Iasi, Romania; 5 Department of Internal Medicine I, “Grigore T. Popa” University of Medicine and Pharmacy, 700115 Iasi, Romania; 6 Department of Inorganic Polymers, “Petru Poni” Institute of Macromolecular Chemistry, 700487 Iasi, Romania; 7 Department of Biophysics and Medical Physics-Nuclear Medicine, “Grigore T. Popa” University of Medicine and Pharmacy, 700115 Iasi, Romania

**Keywords:** Drug delivery, Drug delivery, Drug delivery

## Abstract

The paper aims to investigate the cytotoxic effect on tumor cells of irradiated AuNPs in green light and subsequently functionalized with HS-PEG-NH_2_. The toxicity level of gold conjugates after their functionalization with DOX and TAT peptide was also evaluated. The AuNPs were prepared using the modified Turkevich method and exposed to visible light at a wavelength of 520 nm prior their PEGylation. The optical properties were analyzed by UV-vis spectroscopy, the surface modification was investigated using FTIR and XPS spectroscopies and their sizes and morphologies were evaluated by TEM and DLS techniques. DOX and TAT peptide were linked to the surface of PEGylated AuNPs by reacting their amino groups with glycidyloxypropyl of PEGylated DOX or TAT conjugates under mild conditions at room temperature and in the presence of ethanol as catalyst. The conjugates containing DOX or DOX and TAT have been characterized by fluorescence and FTIR techniques. The changes of electrochemical features were observed using cyclic voltammetry, suggesting a better stability of irradiated nanoparticles. By mass spectrometry it was confirmed that the compounds of interest were obtained. The cell viability test showed that irradiated and non-irradiated nanoparticles coated with PEG are not toxic in normal cells. Tumor cell viability analysis showed that the PEGylated nanoparticles modified with DOX and TAT peptide were more effective than pristine DOX, indicating cytotoxicity up to 10% higher than non-irradiated ones.

## Introduction

The presence of gold nanomaterials (AuNPs) in biomedicine and particularly in antitumor therapy still remains a topic of wide debate, as evidenced by the tremendous amount of scientific works on this issue in recent years^1–3^. An impressive number of research studies have straightened their efforts toward the use of AuNPs in enhancing the efficiency of cancer treatment, due to their ease production and chemical functionalization of their surface^4,5^. Gold nanoparticles are feasible to be developed as versatile nontoxic carriers for drug release as long as they are able to be conjugated with different molecules, including chemotherapeutics, antibodies, peptides, ligands, and other structures which are likely to promote a great capacity to penetrate the tumor site, resulting in a predominant accumulation of bioactive agent in the tumor region^6,7^. On the other hand, the passive anticancer effect based on the accumulation strategy of AuNPs at the tumor site is limited by the inherent heterogeneities of tumor vasculature^8^. It was shown that nanoparticle concentration in the target tissue is influenced by renal clearance rate, and also by activation of immune system mechanisms such as opsonization or nonspecific particle phagocytosis, fulfilled by the reticuloendothelial system (RES).

Different strategies for surface functionalization of AuNPs using a wide range of ligands have been done to overcome these limitations. Polyethylene glycol (PEG) is the polymer known as the most popular material for surface modification in various types of nanoparticulate drugs or gene delivery systems^9–13^. The coverage of the conjugates with PEG moieties plays a major role in improving solubility and stability in aqueous media of the carriers, prolonging the circulation time in the blood stream, bypassing immune recognition due to steric hindrance mechanism^14–16^.

The present work aimed to conduct insightful studies of biological effects starting from previous outcomes involving polymer-coated gold nanoparticles subjected to green light irradiation. According to a previous research^17^ concerning fungal cultures treated with AuNPs suspensions, the first line of defense, expressed by antioxidant enzymes, was quantified by analyzing the activity of superoxide dismutase (SOD), catalase (CAT) and malondialdehyde (MDA) content. The greatest stimulation of CAT and SOD was induced after incubation of cellulolytic fungi cultures with gold nanoparticles irradiated with green light, as compared to similar nanoparticles irradiated with other wavelengths. Since an increased level of ROS has been generated after incubation of fungal cells in the presence of green light irradiated AuNPs, similar irradiated particles are expected to produce comparable effects on human cells, inhibiting the growth of tumor cells.

The nanoparticles were designed to be irradiated just after their stabilization in sodium citrate, for the reason that at this stage the metal atoms could modify their oxidation state with an influence on organic layer conformation^18^. A significant number of works have been concerned about the structure of citrate adlayers on gold nanoparticles, elucidating the binding modes of carboxylate to metallic surface as a consequence of reaction parameters^18–21^. Starting from the classical Turkevich method, the synthesis was enhanced by adding sodium hydroxide to the reaction medium (see Material and Method section), thus obtaining a stable colloidal solution having a concentration ten times higher in gold than in a traditional protocol (from 0.25 to 2.5 mM)^22^. Further polymer coating with PEG, as it can be seen later in this paper, is logically influenced by the oxidation states of gold atoms and also the binding feature between citrate and particle surface^23,24^. The PEGylation using a heterobifunctional polyether derivative was designed to displace the citrate ligand due to formation of stronger Au-S coordinative bonds, although Au-COO^−^ from citrate linkages may also be present, explaining the sporadic occurrence of amine terminal end of PEG in gold surface vicinity, based on electrostatic interaction between -COO^−^ and -NH_3_^+^^25–27^.

A different approach of antitumor therapy was considered in the framework of current research, by which doxorubicin (DOX), one of the most investigated chemotherapeutic agents, was covalently bound to the surface of polymer coated gold nanoparticles^28^. The coupling was envisioned through an oxirane bifunctional linker, able to bind both DOX and PEG due to the presence of a primary amino group^29^. A second important issue was to achieve an efficient penetrability into the tumor cells by the drug loaded particles. Thus, a small number of TAT-peptide grafted onto the polymeric shell has been found as a convenient strategy, with acknowledged results in cellular internalization of non-self-structures and with a highly potential of tumor targeting, as reported in literature^30–32^. FTIR and XPS spectroscopy proved to be of crucial importance in establishing the structure of the final products, with a focus on the differences produced by irradiation. The structure elucidation was completed by morphological and dimensional data performed by TEM and DLS, which reveal uniform entities not exceeding 20 nm in diameter, as disclosed later in this work. The positive ζ potential of the nanoparticles, due to the protonated amino groups onto the polymer coating surface, provide the advantage of being opposite to the cell surface charging, thus facilitating their transfer through cell membranes^33^.

Biological tests have revealed that our drug-free carriers (AuPEG_2000_-NH_2_ and *i*AuPEG_2000_-NH_2_, comprising only the metallic core coated with PEG) do not exhibit cytotoxic effects on normal human dermal fibroblasts and human osteosarcoma. Moreover, the loaded carriers with doxorubicin were more efficient when TAT peptide was attached to the system.

## Results and discussions

### Synthesis

**Figure 1:**
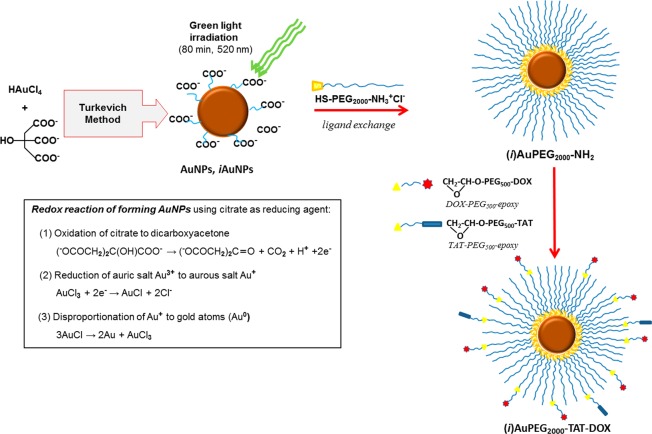
The main steps of AuNPs synthesis followed by surface PEGylation, and conjugation with doxorubicin and TAT peptide.

Following the synthesis protocol described in *Materials and Methods* section and illustrated by Fig. 1, a 2.5 mM suspension of AuNPs was obtained, comprising particles of approximately 17 nm in diameter (16.83 ± 0.25 nm) with long-term stability (over 6 months, determined by macroscopic evaluation and confirmed by DLS and UV-Vis spectroscopy). The enhanced stability of the AuNPs suspension is mainly due to the use of a high pH value during AuNPs formation process in the presence of sodium citrate^22^. It is well known that at high pH, the citrate is fully deprotonated, creating a high abundance of negative charges, inducing repulsions between nearby gold nanoparticles and as a result no aggregation can be obtained.

The *DOX-PEG*_500_*-epoxy* and *TAT-PEG*_500_*-epoxy* precursors were synthesized according to the protocol described in Materials and Methods section (see Supplementary scheme S2 and S3), and characterized by ESI-MS (see Fig. S4 and S5 and section III from Supplementary Information).

### Nanoparticles characterization

By green light irradiation of the nanoparticles, several modifications could be observed regarding the surface properties, compared with non-irradiate ones. These changes were explored both morphologically and structurally, using imaging and spectroscopic techniques, respectively. Consequently, some differences in the oxidation states of the chemical species involved were detected, influencing the chemical bonds established in the nanoparticulate compound. Starting from these findings, our studies further pursued the effect of irradiation of AuNPs on subsequent functionalization as well as their biological activity.

**Figure 2:**
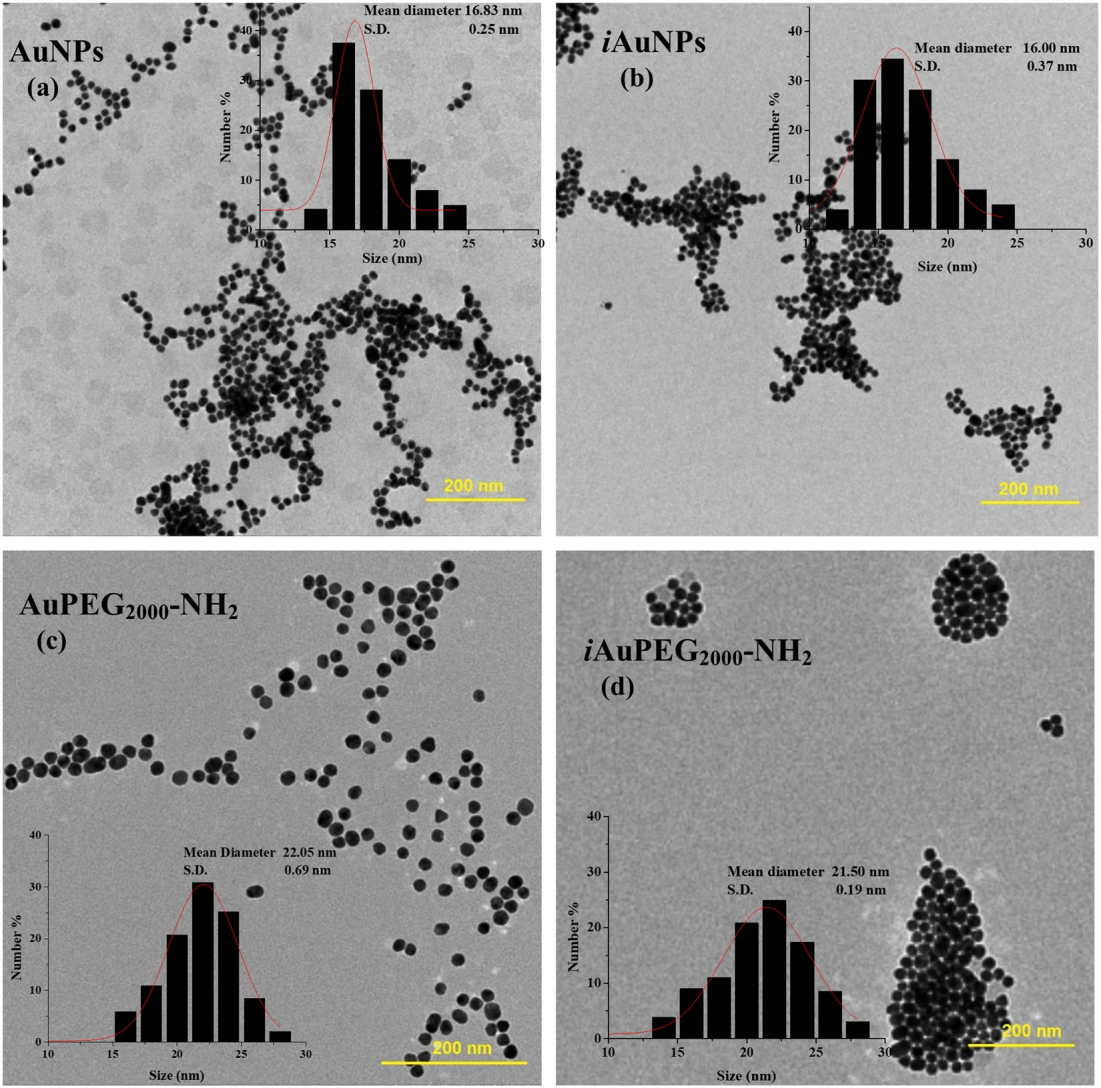
TEM images of non-irradiated particles, (**a**) AuNPs and (**c**) AuPEG_2000_-NH_2_, in comparison with irradiated products, (**b**) *i*AuNPs and (**d**) *i*AuPEG_2000_-NH_2_. The polymer coating has a slight influence on the particle size, but showing a more significant effect on aggregation behavior. The irradiated PEGylated nanoparticles exhibit a phenomenon of uniform clustering, with the formation of entities with dimensions up to 100 nm. The histograms indicating dimensional distribution by measuring about 1000 particles from several TEM images, of which only representative ones are illustrated above.

UV-Vis spectroscopy data, well-known to assess the gold nanoparticle characteristics, principally related to size and concentration, in close connection with imaging data^34,35^, have been detailed and discussed (see Supplementary data, section II.1). TEM imaging data bring essential information regarding the dimension and particle organization. A significant number of images have been acquired and analyzed of which the most representatives are presented below in this paper (Fig. 2); the Fig. S6 in supplementary material comprises overall TEM images of the same compounds, which show more clearly the organization of nanoparticles relative to each other (e.g. clustering, if applicable). TEM micrographs of AuNPs have shown to be predominantly spherical with irregular shapes and displaying a satisfactory dimensional distribution (Figs. 2a and S6a), with a mean diameter of 17 nm. In contrast, *i*AuNPs shows more regular profiles, having a mean diameter of 16 nm, with the tendency to form clusters of undefined shapes (Figs. 2b and S6b). TEM images of PEGylated nanoparticles indicate an increase in particle size (from 16–17 to 22 nm) alongside with a higher level of clustering^36^. As revealed by the Fig. 2d, the irradiated particles (*i*AuPEG_2000_-NH_2_), illustrates an agglomeration tendency, as compared to the non-irradiated ones (Fig. 2c), but in the first case the entities are arranged in groups with regular forms of less than 80 nm in diameter. The formation of these small associations was also confirmed by DLS investigations, where the measured values for the hydrodynamic diameter disclose the presence of two populations: one with tens of nm values representing the free particles, and another around 400 nm representing clustered particles (see Supplementary Table S3). The agglomeration trend in the AuPEG_2000_-NH_2_/*i*AuPEG_2000_-NH_2_ compounds can be explained either by the occurrence of the S-S cross-linking between the polymer chains at 22 °C, but also due to an increase in ζ potential from –40 mV in AuNPs to about +28 mV after PEGylation^37^.

The hydrodynamic diameter of nanoparticles obtained by DLS measurements along with their ζ potential offer notable information regarding the spatial distribution of global electrical charge, indicating different ways of organizing the polymeric layer in irradiated entities as compared to non-irradiated ones. As a result, the average hydrodynamic diameter of AuNPs was found of 39 nm, significant higher than *i*AuNPs, having a mean diameter of 19.5 nm, although the TEM images indicate only small differences between the two products (see Supplementary Table S3). This outcome was explained by significant higher ζ potential values, of around –40 mV in AuNPs, than in *i*AuNPs (–30 mV), suggesting a concentration of -COO^−^ groups with higher probability at a longer distance from metallic atoms in the case of non-irradiated particles^18^. In correlation with XPS and electrochemistry data, we can assess that by green light irradiation the metallic core contain a higher amount of Au^+^ species (over 15%) which are mainly involved in linkages between citrate and metallic atoms. After PEGylation, we can observe an increase in the ζ potential value of irradiated compounds (+29.07 mV) compared to the non-irradiated ones (+26.97 mV), with an insignificant difference in the hydrodynamic diameters of 44.9/43.3 (see Table additional S3). Accordingly, the XPS results showed a smaller concentration of both COOH and COO^**−**^ groups in *i*AuPEG-NH_2_, with a higher amount of C-C/C-N, C-N and C-S than in AuPEG_2000_-NH_2_ (see Supplementary Table S5). All these outcomes suggest that in the case of irradiated particles the ligand exchange between citrate and HS-PEG_2000_-NH_2_ took place in a greater extent as compared to non-irradiated ones, while a number of citrate molecules remains bound to metal atoms, among the PEG chains^18^. According to^36^ when AuNPs are exposed to green light irradiation a tendency of clusterization of the nanoparticles can be observed. As a consequence, good particle stability was achieved following the illumination process, as ζ potential measurements indicate and also confirmed by cyclic voltammetry.

FTIR spectroscopy, in close correlation with XPS data, highlighted the structure differences between the irradiated compounds versus non-irradiated ones, which originate in the type of interaction between citrate ions and gold atoms, more precisely concerning the conformation adopted by the citrate molecules involved in gold nanoparticle stabilization. The spectra of all intermediate products (AuNPs, *i*AuNPs, AuPEG_2000_-NH_2_ and *i*AuPEG_2000_-NH_2_) are presented and extensive discussed in section IV, Supplementary Information available.

**Figure 3:**
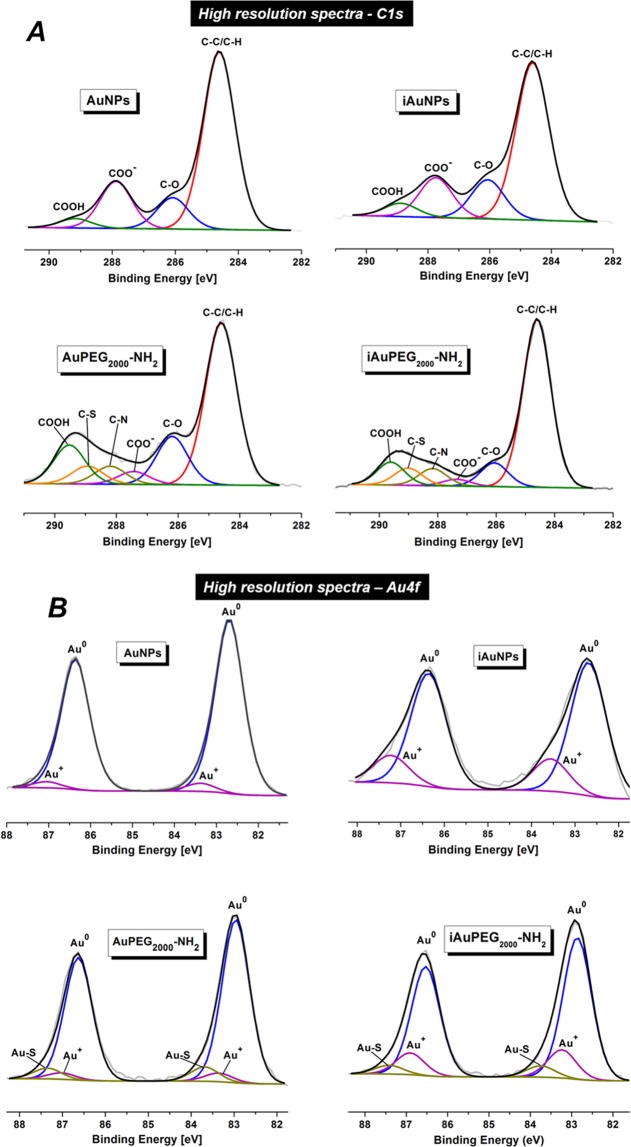
XPS high resolution spectra (**A**) C1s and (**B**) Au4f signals of AuNPs, *i*AuNPs, AuPEG_2000_-NH_2_ and *i*AuPEG_2000_-NH_2_.

Furthermore, XPS analysis was also carried out to investigate the surface oxidation states and chemical composition of the irradiated compounds versus non-irradiated ones. The C1s signal in the AuNPs suggests that COO^−^ groups bind to gold by two coordination modes: η2 Au-COO (chelating) at 286.7 eV and η1 Au-COO^−^ (bridging) at 288.1 eV. In *i*AuNPs the COO^−^ group binds to the gold surface by a single η1 Au-COO^−^ (chelating) coordination mode at 288.2 eV. The high-resolution spectra C1s (Fig. 3A) revealed the presence of C-H/C-C, C-O, C-N, C-S, COO^−^ and COOH bonds at 284.6, 286.2, 288.2, 289, 284.4 and 289.5 eV for both AuPEG_2000_-NH_2_ and *i*AuPEG_2000_-NH_2_ compounds^23^. The lower percentage of C-O bond, correlated to the smaller percent of COO^−^ and COOH, in *i*AuPEG_2000_-NH_2_ than in AuPEG_2000_-NH_2_, suggests that the amount of remaining citrate is significantly reduced in the case of irradiated nanoparticles. Additionally, in the *i*AuPEG_2000_-NH_2_ conjugates, the C1s spectrum confirmed the presence of the bridging mode η1 Au-COO^−^ and the disappearance of the chelation mode (Fig. 3A).

The Au4f high-resolution spectra of the AuNPs (Fig. 3B) revealed two pair of peaks: one at 82.7 and 86.4 eV which are due to elemental gold (Au°), while the pair at 83.4 and 87.1 eV is due to Au^+^ species. Larger peaks at 82.9 and 86.6 eV in AuPEG_2000_-NH_2_ were assigned to Au° and smaller peaks at higher energies (83.7 and 87.4 eV) were attributed to the chemical link between thiol groups of PEG and gold (Au-S). Similarly, in the case of irradiated nanoparticles, *i*AuNPs, the first pair of peaks at 82.7 and 86.4 eV is due to Au°, while the second one at 83.6 and 87.3 eV was assigned to Au^+^ species. The PEG chains did not change the position of the pair corresponding to Au° in both irradiated or non-irradiated nanoparticles. The *i*AuPEG_2000_-NH_2_ product revealed a slightly smaller percent of Au-S bond as compare to AuPEG_2000_-NH_2_ (6.23% ± 1.36 *vs*. 7.72% ± 1.62), while Au^+^ percent still keeps a substantial gap of over 10% against its non-irradiated analogous (15.63% ± 1.82 *vs*. 4.62% ± 2.35) (see Supplementary Table S5). On the other hand, the C1s spectra indicate that the percent of C-S bond (directly related to the number of PEG chains) is almost the same in both products, slightly higher in irradiated ones (6.73% ± 0.83 *vs*. 6.03% ± 1.00). These findings suggest that the polymer coating of irradiated particles, in a similar extent as non-irradiated ones, given by the C-S percent, was more strongly linked to the metallic surface via Au-S bonds, due to the large amount of Au^+^ species, besides other electrostatic interactions, as high resolution spectra Au4f indicates, thus explaining the remarkable stability of irradiated nanoparticles over time as compared to non-irradiated ones.

To obtain AuPEG_2000_-DOX, *i*AuPEG_2000_-DOX, AuPEG_2000_-TAT-DOX and *i*AuPEG_2000_-TAT-DOX conjugates, the amino terminal groups of AuPEG_2000_-NH_2_ or *i*AuPEG_2000_-NH_2_ were reacted with epoxy groups of DOX-PEG_500_-epoxy and/or TAT-PEG_500_-epoxy (see Supplementary Scheme S1 and S2) water solutions in the presence of isopropanol (as catalyst) in a molar ratio OH: epoxy groups added in the system was 1: 1, according to literature data^38^. The grafting reactions were carried out taking into account the molar ratio between the participating molecular species HS-PEG_2000_-NH_2_: DOX-PEG_500_-epoxy: TAT-PEG_500_-epoxy of 10: 1: 0.1 molar ratio which is equivalent to the ratio Au: DOX-PEG_500_-epoxy: TAT-PEG_500_-epoxy of 1: 2.3: 0.23. It should be noted that the HS-PEG_2000_-NH_2_ is linked to the AuNPs surfaces and it was considered that the ratio of 1: 20 between gold and HS-PEG_2000_-NH_2_ remained unchanged after purification of AuPEG_2000_-NH_2_ or *i*AuPEG_2000_-NH_2_ conjugates. The mixtures were maintained under stirring at 18 °C for 72 hours. As it was mentioned in Materials and Methods section, DOX-PEG_500_-epoxy conjugates were used without purification in subsequent reactions with AuPEG_2000_-NH_2_ or *i*AuPEG_2000_-NH_2_ nanoparticles, meaning that the small amount of free doxorubicin added together with DOX-PEG_500_-epoxy conjugates can involve many hydrogen bonds with donor/acceptor moieties due to the presence of multiple hydroxyl and carbonyl groups in its composition, making possible its stabilization onto polymer chains surrounding the newly formed nanoconjugates, expressed by the codes: AuPEG_2000_-DOX, *i*AuPEG_2000_-DOX, AuPEG_2000_-TAT-DOX and *i*AuPEG_2000_-TAT-DOX. This finding was demonstrated before by Hutchins^39^ when DOX formed hydrogen bonds with PEG moiety from PE-b-PEG copolymers. Under this circumstance, the “free” DOX is assumed to leave the carrier in the first step, prior to release of the drug in major amount, which was covalently bound onto the polymer layer of the gold nanoparticles.

**Figure 4:**
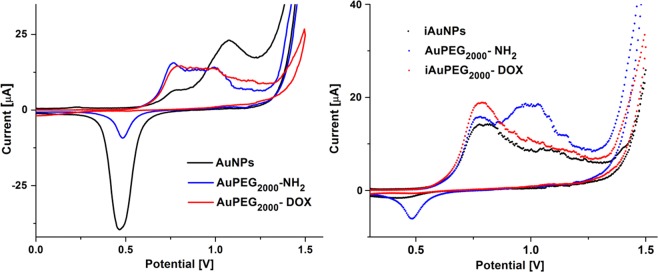
Cyclic voltammograms of non-irradiated: AuNPs, AuPEG_2000_-NH_2_ and AuPEG_2000_-DOX (**a**); and irradiated samples: *i*AuNPs, *i*AuPEG_2000_-NH_2_ and *i*AuPEG_2000_-NH_2_ (**b**), sweeping the potential between −1 to 1.5 V, at 100 mV·s^–1^ in 0.05 M H_2_SO_4_.

The redox behavior of non-irradiated gold nanoparticles (AuNPs, AuPEG_2000_-NH_2_ and AuPEG_2000_-DOX) deposited onto electrodes was compared with that of irradiated analogues (*i*AuNPs, *i*AuPEG_2000_-NH_2_ and *i*AuPEG_2000_-DOX) (Fig. 4). The cyclic voltammetry of non-irradiated AuNPs (Fig. 4a) shows two anodic peaks located at +0.77 V ascribed to the oxidation of metallic gold to gold oxide (Eq. 1) and the second peak at +1.07 V due to the oxidation of gold to the trivalent state (Eq. 2)^40,41^.

1 $$2{{\rm{Au}}}^{0}+{{\rm{H}}}_{2}{\rm{O}}\to {{\rm{Au}}}_{2}{\rm{O}}+2{{\rm{H}}}^{+}+2{{\rm{e}}}^{-}$$

2 $${{\rm{Au}}}^{0}+3{{\rm{H}}}_{2}{\rm{O}}\to {\rm{Au}}{({\rm{OH}})}_{3}+3{{\rm{H}}}^{+}+3{{\rm{e}}}^{-}$$

Anodic processes are accompanied by one cathodic peak around +0.47 V in the reverse scan, associated to the reduction of gold oxide species back to metallic gold, especially with the reduction of Au^3+^ ^42^. The same oxidation peaks are also observed for *i*AuNPs (Fig. 4b), with an enhanced oxidation current for the second peak located at +0.77 V and less defined oxidation peak at +1.07 V, which can be related to the strong effect of irradiation which creates more sites prone to first electronic transfer. The oxide film which is formed in the positive scan is not entirely reduced in the reverse scan as seen by the smaller reduction peak. This indicates that higher energy is required for its reduction compared to non-irradiated nanoparticles. By analyzing cyclic voltammetry, it is highlighted that for non-irradiated gold nanoparticles there is an increase in oxygen binding to two gold atoms followed by their reduction, while for irradiated nanoparticles this reduction of oxide film is almost vanished, suggesting a better stability of irradiated nanoparticles. The electrochemical properties of AuNPs are considerably diminished by PEG coating and further by coverage with DOX. It has also been observed a slight increase of current for the first oxidation peak when particles are covered by DOX, which is attributed to the redox activity of DOX (which shows oxidation at the same potential, data not shown). The lack of reduction peaks for AuPEG_2000_-DOX and *i*AuPEG_2000_-DOX might be attributed to the passivation of Au nanoparticles with a monolayer of DOX.

**Figure 5:**
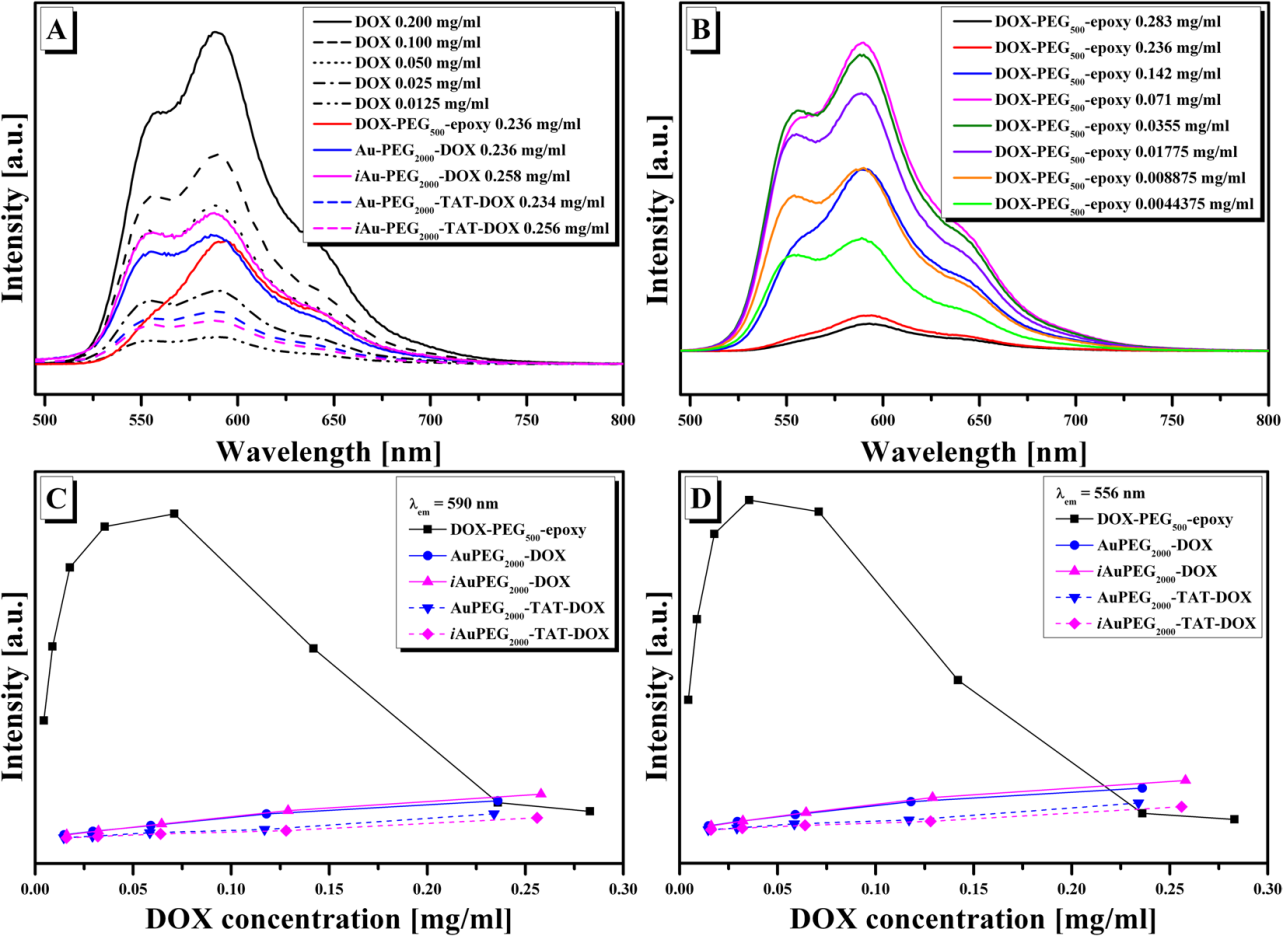
(**A**) The fluorescence emission spectra of pristine DOX at different concentrations and of DOX-PEG_500_-epoxy, AuPEG_2000_-DOX, *i*AuPEG_2000_-DOX, AuPEG_2000_-TAT-DOX, *i*AuPEG_2000_-TAT-DOX at DOX concentration of around 0.2 mg/mL; (**B**) the fluorescence emission spectra of DOX-PEG_500_-epoxy at different DOX concentrations; (**C, D**) the variation of the fluorescence peaks *vs*. DOX concentration of the studied systems at λ_em_ = 590 nm and λ_em_ = 556 nm respectively; all spectra were registered at λ_ex_ = 480 nm.

Figure 5 presents the fluorescence emission spectra of pristine DOX and of conjugates at excitation wavelength λ_ex_ of 480 nm. The fluorescence emission spectra of pristine DOX as a function of its concentration present two characteristic emission peaks at 556 and 590 nm with decreasing in their intensities once the concentration of DOX decreases (Fig. 5A). From the same figure it can be observed that the fluorescence intensities of all studied conjugates have decreased compared to those of pristine DOX at the same DOX concentration in the series: DOX > *i*AuPEG_2000_-DOX > AuPEG_2000_-DOX > DOX-PEG_500_-epoxy> AuPEG_2000_-TAT-DOX > *i*AuPEG_2000_-TAT-DOX, leading to the idea that with the increase of PEG concentration in the system the quenching effect on DOX photoluminescence is more pronounced. In this idea, the emission spectra *vs*. the concentration of DOX-PEG_500_-epoxy intermediate were registered (Fig. 5B). From this figure and from the variation of the peak intensities at λ_em_ = 590 nm (Fig. 5C) or λ_em_ = 556 nm (Fig. 5D) as a function of PEG concentration, the intensities of the peaks increased up to a certain concentration and then began to decrease with further increase in PEG concentration along with that of DOX. In this context, we can conclude that the presence of PEG in the DOX-PEG_500_-epoxy solutions manifests a quenching effect on DOX, depending on PEG concentration, which indicate on the one hand that DOX was bounded to PEG molecule^43^ and on the other hand, being also suitable to involve hydrogen bonds with PEG moieties^44^. The emission intensities of DOX-PEG_500_-epoxy, AuPEG_2000_-DOX and *i*AuPEG_2000_-DOX solutions at higher DOX concentration (around 0.2 mg/mL) are in the same range (Fig. 5A), deducing the fact that the PEG moieties from DOX-PEG_500_-epoxy precursor has a major influence in the decreasing of DOX photoluminescence. This remark was not sustained when we decreased the concentration of DOX together with that of Au conjugates from the studied solutions (Fig. 5C,D), in fact irradiated or non-irradiated Au-PEG conjugates together with PEG from DOX-PEG conjugates contributed to the quenching effect of DOX photoluminescence. Also, it should be added that fluorescence quenching effect on DOX is more pronounced after the addition of TAT to either irradiated or non-irradiated conjugates (Fig. 5A,C,D).

**Figure 6:**
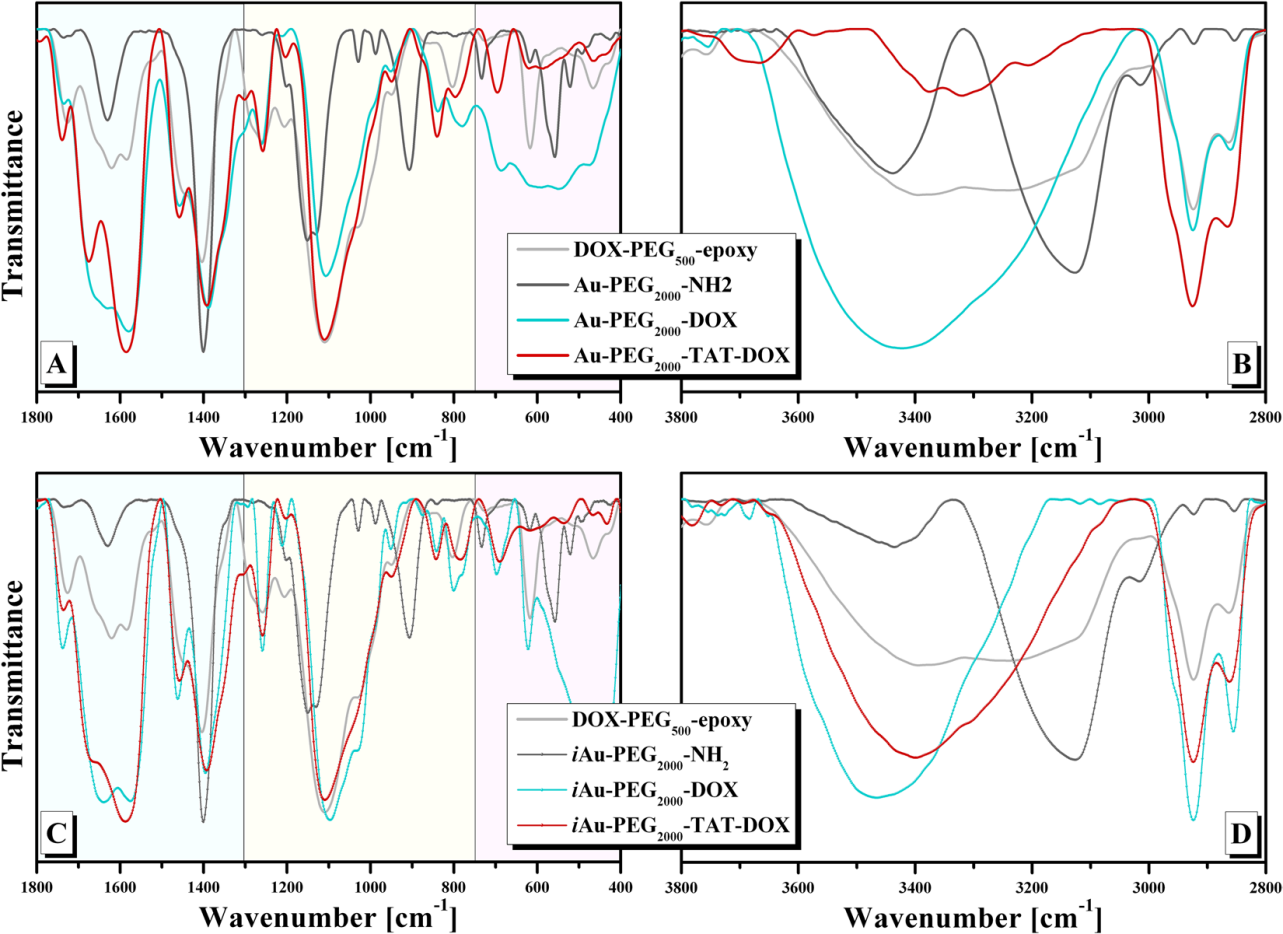
FTIR spectra of: (**A, B**) AuPEG_2000_-DOX, AuPEG_2000_-TAT-DOX and (C, D) *i*AuPEG_2000_-DOX, *i*AuPEG_2000_-TAT-DOX conjugates along with that of DOX-PEG_500_-epoxy, AuPEG_2000_-NH_2_ and *i*AuPEG_2000_-NH_2_ intermediates.

The fluorescence emission spectra of AuPEG_2000_-DOX, *i*AuPEG_2000_-DOX, AuPEG_2000_-TAT-DOX, *i*AuPEG_2000_-TAT-DOX conjugates do not give information about the composition of the conjugates, giving information about the behavior of DOX (as fluorescence probe) in different microenvironments^45^. Because of this reason FTIR spectra are necessary to confirm the presence of ligands on AuNPs layers. Figure 6 presents the FTIR spectra of AuPEG_2000_-DOX*, i*AuPEG_2000_-DOX, AuPEG_2000_-TAT-DOX, *i*AuPEG_2000_-TAT-DOX conjugates along with that of DOX-PEG_500_-epoxy, AuPEG_2000_-NH_2_ and *i*AuPEG_2000_-NH_2_ intermediates. The spectra were normalized taking into consideration the band of 1390 cm^−1^ (C-H bending vibration) which does exist in all represented spectra. The spectra shown four major characteristic regions: the first (400–750 cm^−1^) is related to AuNPs bonding with sulfur (Au-S), remaining oxygen from citrate, and C-S from HS-PEG_2000_-NH_2_; the second one (750–1300 cm^−1^) is attributed to C-O stretching vibrations; the third region (1300–1800 cm^−1^) is represented by C=O stretching, amide I (stretch), amide II and III, C=C stretch and C-H bending and the last one (2800–3600 cm^−1^) is characteristic for O-H, N-H and C-H stretching vibrations. The FTIR spectra of AuPEG_2000_-DOX and *i*AuPEG_2000_-DOX present DOX characteristic bands in 1100–1800 cm^−1^ interval, and are sifted to those in DOX-PEG_500_-epoxy and AuPEG_2000_-NH_2_, being a sign that there are interactions between AuPEG_2000_-NH_2_ and DOX-PEG_500_-epoxy^46^. The region of 3400 cm^−1^ was not discussed because there are overlapping of specific characteristic bands of O-H and NH stretching vibrations. The bands at 2924 and 2860 cm^−1^, specific for C-H stretching vibrations, are more intense and better resolved.

In the FTIR spectra of AuPEG_2000_-TAT-DOX, *i*AuPEG_2000_-TAT-DOX the bands at 1676 (amide I), 1583 cm^−1^ (amide II) and 1392 (amide III) or 1668, 1587 and 1392 cm^−1^ respectively are well resolved with their intensity higher than 1, meaning the capping of peptide molecules on the surface. All assignments of bands are presented in Table S4 from Supplementary Information available.

### Biological assay

The final compounds AuPEG_2000_-DOX, *i*AuPEG_2000_DOX, AuPEG_2000_-TAT-DOX and *i*AuPEG_2000_-TAT-DOX were subjected to biological testing as they resulted from the last synthesis step, without further purifications. This strategy relies on the fact that unreacted precursors DOX, cys-TAT, DOX-PEG_500_-epoxy and TAT-PEG_500_-epoxy may be physically adsorbed on gold surface due to the presence of NH_2_ groups, or among PEG chains that are able to engage in hydrogen bonding^44^.

**Figure 7:**
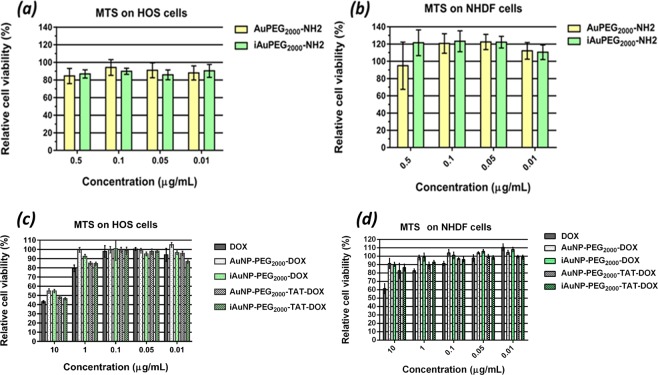
Concentration dependence of cytotoxicity of carriers on (**a**) HOS cells and on (**b**) NHDF cells. Concentration dependence of cytotoxicity of carriers loaded with doxorubicin on (**c**) HOS cells and (**d**) NHDF cells.

To evaluate the toxicity of the drug-free carriers (AuPEG_2000_-NH_2_ and *i*AuPEG_2000_-NH_2_), we have used a metabolic assay (MTS) which measures the mitochondrial reductase activity in HOS or NHDF cells incubated with the irradiated and non-irradiated carrier at specific concentrations (0.5, 0.1, 0.05 and 0.01 μg/mL) as revealed by Fig. 7. Initially, volumes of 1000 µL stock solution of 1 mg/mL were prepared starting from both AuPEG_2000_-NH_2_ and *i*AuPEG_2000_-NH_2_ suspensions as obtained after the removal by centrifugation of the HS-PEG_2000_-NH_2_ excess, having the same concentration of 8.04 μg/mL, calculated by taking into account the concentration of Au and HS-PEG_2000_-NH_2_, 0.2·10^−3^ mM and 4·10^−3^ mM, respectively, which corresponds to 0.0394 and 8 mg existing in 1000 µL (as calculated and presented in Supplementary Information file). Successive dilutions have been made afterwards, in order to reach the above-mentioned concentrations, which were further subjected to cytotoxicity assay.

Cells with healthy mitochondrial function could reduce tetrazolium salt to formazan and as a result, the mitochondrial reductase activity is used as an indirect indicator for cell viability. As shown in Fig. 7b, the treatment of all compounds in concentrations of up to 0.5 μg/mL did not reduce the viability of NHDF cells after 48 h of treatment, whereas the same concentrations of compounds caused a slight reduction in the HOS cell survival as can be seen in Fig. 7a. Analyzing comparatively the results obtained on the two cell types, we can infer that carriers *per se* are non-cytotoxic, with a pronounced proliferation effect when normal cells are involved^47^.

Irradiated and non-irradiated carriers loaded with doxorubicin were tested on HOS cells in two forms: enhanced with TAT peptide and without peptide (Fig. 7c). Naked doxorubicin was used as control, in the same concentrations as drug loaded nanoparticles: 10, 1, 0.1, 0.05 and 0.01 µg/mL.

The samples comprising doxorubicin were prepared at 100, 10, 1, 0.1, 0.05 and 0.01 µg/mL as presented in Section VI from Supplementary Information, available.

**Figure 8:**
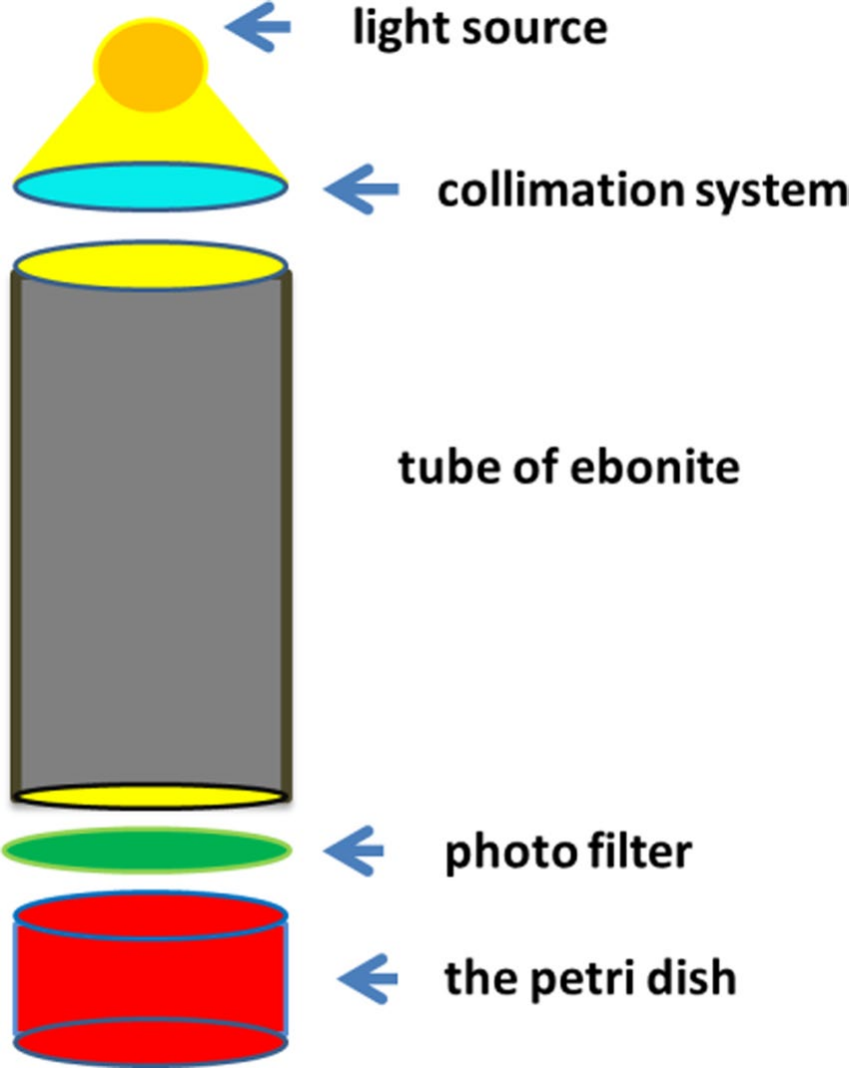
The schematic representation of the illumination system used to irradiate the gold nanoparticle suspension samples.

Analyzing the results obtained by MTS assay, synthesized by Fig. 8c, one can observe that AuPEG_2000_-TAT-DOX and *i*AuPEG_2000_-TAT-DOX are slightly more active than AuPEG_2000_-DOX and *i*AuPEG_2000_-DOX.

TAT is a cell-penetrating peptide. Cell-penetrating peptides are usually short peptides which cross cellular membrane and could be used to facilitate the cellular uptake of various molecular cargoes which are otherwise hard to transfer inside the cytoplasm^48^. Increased activity of compounds coupled with TAT could be explained by the ability of this peptide to enhance cell penetration.

A second observation is an enhanced effect, but not significant of irradiated compounds *i*AuPEG_2000_-DOX and *i*AuPEG_2000_-TAT-DOX at 1 µg/mL and 0.01 µg/mL compared to non-irradiated forms.

Regarding the overall activity of our gold nanoparticles loaded with doxorubicin, since 10 µg/mL of AuPEG_2000_-TAT-DOX contains less than 0.3 µg/mL of pure doxorubicin (see Materials and Methods section), we could infer that they are more active than doxorubicin alone. More specifically, when treating cells with 10 µg/mL pure doxorubicin, the cell viability is very close to that encountered in cells treated with a suspension of 10 µg/mL drug loaded nanoparticles: 42% ± 2.1 versus 47% ± 2.6, respectively. This finding means that the conjugate *i*AuPEG_2000_-TAT-DOX exerts approximately the same effect as the pure drug, but using a 97% lower amount of DOX. The same doxorubicin conjugates tested on NHDF cells exhibited a similar behavior with a slightly lower cytotoxicity (Fig. 7d).

## Conclusion

The present research aimed to design an antitumor agent based on gold nanoparticles with superior efficacy in the context of minimal drug loading, in order to avoid adverse effects on healthy tissues, and ensuring at the same time an acceptable colloidal stability. The gold nanoparticles stabilized by sodium citrate were irradiated in visible light at 520 nm before being coated with polyethylene glycol. Both macroscopic examination and surface analysis have shown that the irradiation enhanced the particle stability even at higher concentrations, keeping a narrow dimensional distribution. Advanced structural characterization techniques highlighted several differences between irradiated products against the non-irradiated ones, which can explain the colloidal behavior of the particles, their stability, and also the auspicious antitumor activity, as reported by literature data. The resulting structures may have the potential to avoid systemic toxicity and side effects on healthy tissues when used as drug carriers, if taken into account the viability of normal versus malignant cells under the influence of unloaded nanoparticles. Another reason for such favorable results, with a great potential for deepened research, is the covalent binding of doxorubicin, leading to a new molecule that may act differently, in a synergistic way with the free drug adsorbed into the coating. Under the conditions of present study, the main achievement is that a similar cytotoxic effect has been obtained on malignant HOS cells when using only 3% doxorubicin (loaded into nanoparticles) as compared to pure drug treated cells.

## Materials and Methods

### Materials

Gold(III) chloride trihydrate (HAuCl_4_·3H_2_O, M_w_ = 393.83 g/mol), sodium hydroxide (NaOH), sodium citrate (Na_3_C_6_H_5_O_7_·2H_2_O), polyethylenglycol diglycidyl ether (PEG_500_, M_n_ ~ 500 g/mol) and doxorubicin (DOX, Mw = 579.98 g/mol) were purchased from Sigma Aldrich. Thiol PEG amine hydrochloride (HS-PEG_2000_-NH_2_·HCl, Mn ~ 2000 g/mol) was obtained from JenKen Technology USA, and cysteine-terminated TAT peptide (TAT-cys, Mw = 1315 g/mol) of 95% purity (GRKKKRRQRC) from ChemPeptide Limited company.

### Cell culture

Human osteosarcoma (HOS) and Normal Human Dermal Fibroblasts (NHDF) cell lines were purchased from CLS Cell Lines Service GmbH and PromoCell, Eagle’s minimal essential medium (MEM) from Lonza, fetal bovine serum (FBS) from Biochrom GmbH, Germany and 1% Penicillin-Streptomycin-Amphotericin B mixture (10 K/10 K/25 µg in 100 ml) from Lonza, CellTiter 96® Aqueous One Solution Cell Proliferation Assay from Promega, Trypsin-Versene (EDTA) mixture from Lonza, phosphate buffered saline (PBS) from Invitrogen.

### Synthesis of PEGylated gold nanoparticles conjugated with doxorubicin

The main synthesis steps are detailed as follows, being presented by the overall reaction scheme (Fig. 1) comprising both formation and stabilization of the particles with citrate, and then PEG, as well as the particle conjugation with TAT peptide and doxorubicin by covalent coupling.

### Synthesis of citrate stabilized gold nanoparticles (AuNPs)

In a preceding stage, Stock solutions of HAuCl_4_ (25 mM) and NaOH (20 mM) in deionized water were prepared. The gold nanoparticles (AuNPs) with a reproducible size of 17 nm were synthesized using the modified Turkevich method^49^, gaining a narrow size distribution by adding sodium hydroxide into the reaction mixture as reported by Li *et al*.^22^. Briefly, in a round flask of 100 mL, immersed in a heated water bath with magnetic stirrer, 5 mL HAuCl_4_ 25 mM (0.125 mmol) and 16.5 mL NaOH 20 mM (0.33 mmol) were added and the final volume was adjusted up to 50 mL with deionized water to obtain a solution of 2.5 mM HAuCl_4_. The mixture was heated for 30 min., when the system has reached 90 °C. In the magnetic stirring conditions, 1.5 mL of sodium citrate (50 mM) was added and the heat source was maintained a further 15 min. The red wine suspension was cooled to 4 °C to prevent aggregation or growth of the AuNPs.

### Exposure of AuNPs to visible light

The nanoparticle irradiation was performed using an illumination system comprising of an ebonite tube of 24.5 cm length, equipped with a light source (a 50 W helium lamp) and a collimation system, at its upper end, and with a green photo filter positioned at the other end of the same tube (Fig. 8). 10 mL of the prepared AuNPs suspension (2.5 mM) were added in a Petri dish (having the same diameter as ebonite tube, 38 mm) in order to be irradiated. The sample was exposed to green light in four cycles of 20 min lighting between 5 min darkness – to keep the sample temperature around 25 °C. The illumination level of irradiated samples was done with Solar Light PMA 2100 device, equipped with PMA 2130 sensor. The primary energy emitted by the source was 20.6 W/m^2^, corresponding to an energy absorbed of 3.22 kJ/m^2^.

### Synthesis of PEGylated gold nanoparticles

#### PEGylation of the non-irradiated AuNPs (AuPEG_2000_-NH_2_)

 Ten mL of 0.25 mM non-irradiated AuNPs suspension (2.5·10^−3^ mmol), obtained by 1:10 dilution with ultrapure water (0.05 µS·cm^−1^) of the 2.5 mM AuNPs suspension, were mixed with 100 mg HS-PEG_2000_-NH_2_^.^HCl (0.05 mmol) in order to gain a ratio between thiol ligand and gold of 20:1. The mixture was kept under vigorous magnetic stirring for 24 h. The resulted suspension was sonicated for 10 min on bath water (mild conditions) and then centrifuged for 60 min at 5000 rpm to remove the thiol ligand excess and citrate. The supernatant was collected and replaced with a similar volume of deionized water. This purification procedure was repeated two times, followed by resuspension of PEGylated nanoparticles in deionized water to reach a final volume of 10 mL. After particle purification about 80% of AuPEG_2000_-NH_2_ suspension was recovered, having a concentration of 0.2 mM in gold. This percent was established based on UV-Vis measurements. A calibration curve was plot measuring the absorbance of known concentrations of unpurified AuPEG_2000_-NH_2_ between 0.25 and 0.02 mM (see Supplementary Information) and after that, using the line equation and measuring the absorbance of resulting solution at 536 nm (0.838 a.u.), the concentration of purified product was determined.

#### PEGylation of the irradiated AuNPs (*iAuPEG*_2000_*-NH*_*2*_*)*

 Ten mL of 0.25 mM irradiated *i*AuNPs suspension (2.5·10^−3^ mmol) was subjected to the same procedure as in the above paragraph, using the same ratio between HS-PEG_2000_-NH_2_^.^HCl and Au of 20:1. 10 mL *i*AuPEG_2000_-NH_2_ suspension were obtained, reaching a concentration of about 0.2 mM after purification, determined from UV-Vis data, using a similar reasoning as in non-irradiated product (see the calibration curve, Supplementary Information). At 528 nm, the measured absorbance of purified product was 1.421 a.u., corresponding to a concentration of about 0.2 mM in gold.

### PEGylation of DOX (DOX-PEG_500_-epoxy)

The covalent binding of DOX with AuNPs took place using PEG_500_, as a bifunctional linker, able to bind NH_2_ from DOX at one end and NH_2_ from PEG of AuNPs coating at the other end, via oxirane opening ring (Scheme S1, SI). A stock solution of 5 mg/mL DOX was prepared by dissolving 4.6 mg DOX in 920 μL ultrapure water and then stored at 4 °C. 2.58 mg PEG_500_ (5.16·10^−3^ mmol) were added over 600 μL DOX stock solution (3 mg, 5.17·10^−3^ mmol) to get a molar ratio PEG_500_: DOX of 1: 1. This ratio allowed the formation of DOX-PEG_500_-epoxy product, keeping an unreacted oxirane group. 2 μl of isopropyl alcohol were also added to catalyze the oxirane cycle opening by amino groups. The reaction mixture was maintained under continuous stirring, with the help of a thermomixer set at 500 rot/min and 22 °C, for 72 hours. Completion of the reaction was confirmed by mass spectrometry as depicted in paragraph 2.4.5.

### PEGylation of TAT (TAT-PEG_500_-epoxy)

A stock solution of 10 mg/mL cys-TAT was prepared by dissolving 10 mg peptide in 1000 μL ultra-pure water to be stored at at 4 °C. A volume of 263 μL cys-TAT stock solution (2.63 mg) and 2 μL isopropyl alcohol were added to 100 μL aqueous solution of 10 mg/mL PEG_500_ (1 mg) to get a molar ratio PEG_500_: cys-TAT of 1: 1. For this ratio, the TAT-PEG_500_-epoxy product was expected to contain one free oxiranic ring. The resulted mixture was maintained for 72 hours, under continuous stirring, using a thermomixer set at 500 rot/min and 18 °C. Completion of the reaction was confirmed by mass spectrometry as depicted in paragraph 2.4.5.

### ***Conjugation of AuPEG***_***2000***_***-NH***_**2**_***and iAuPEG***_**2000**_***-NH***_**2**_***with DOX-PEG***_**500**_***-epoxy and TAT-PEG***_**500**_***-epoxy***

Reaction of amine groups of *AuPEG*_2000_*-NH*_2_ *and iAuPEG*_2000_*-NH*_2_ *with epoxy groups of DOX-PEG*_*500*_*-epoxy and TAT-PEG*_*500*_*-epoxy* precursors (see Scheme 1) was conducted in presence of isopropanol for 72 h at 18 °C Briefly, in 2 mL microcentrifuge tubes were added volumes of 1000 μL AuPEG_2000_-NH_2_ or *i*AuPEG_2000_-NH_2_ (comprising 0.2·10^−3^ mmol gold and 4·10^−3^ mmol PEG2000), 50 μL DOX-PEG_500_-epoxy (0.46·10^−3^ mmol) and 8.33 μL TAT-PEG_500_-epoxy (0.046 ·10^−3^ mmol) and isopropanol in a molar ration of OH: mmol added epoxy groups = 1: 1^38^. The mixtures were maintained under stirring, with the help of a thermomixer set at 500 rot/min, for 72 hours at 18 °C. The samples were used for biological tests without purifications and were noted as AuPEG_2000_-TAT-DOX and *i*AuPEG_2000_-TAT-DOX, respectively.

Two additional batches of drug-loaded nanoparticles were obtained, without TAT-PEG_500_-epoxy grafted on the surface, using the same strategy as before but without the presence of TAT-PEG_500_-epoxy conjugate. The samples were noted as AuPEG_2000_-DOX and *i*AuPEG_2000_-DOX.

AuPEG_2000_-DOX, *i*AuPEG_2000_-DOX, AuPEG_2000_-TAT-DOX and *i*AuPEG_2000_-TAT-DOX conjugates were submitted for biological tests to reveal their actions on antitumor activity.

### Structural characterization

#### Fourier-transform infrared spectroscopy (FTIR)

FTIR spectra were obtained in transmission mode using a Bruker Vertex instrument, model 70. The samples were prepared by depositing the nanoparticle suspension on KBr pellets which were then subjected to a drying process (using a UV lamp) before recording the spectra. The spectra ranged from 4000 to 400 cm^−1^ with a resolution of 2 cm^−1^.

#### X-ray Photoelectron Spectroscopy (XPS)

XPS data were achieved on an Axis NOVA instrument (Kratos Analytical, Manchester, United Kingdom), using AlKα (1486.6 eV) as X-ray source, with 20 mA current and 15 kV voltages (300 W), under a base pressure of 10^−8^÷10^−9^ Torr in the sample compartment. The incident monochromatic X-ray beam was focused on a 0.7 mm ×0.3 mm XPS area of the sample surface. The high-resolution spectra for all the elements of interest were the average of five scans acquired using a pass energy of 20 eV and a step size of 0.1 eV. The binding energy of the C 1 s peak, normalized at 284.6 eV, has been established as reference value for all binding energies. XPS data fitting was accomplished using the ESCApe software, by applying Gaussian-Lorentzian mixed function.

#### Florescence spectroscopy

Fluorescence measurements were carried out using a FluoroMax-4 spectrophotometer (Horiba, Kyoto, Japan). The emission spectra were collected using an excitation wavelength of 480 nm.

### Particles size and morphology

#### Transmission electron microscopy (TEM)

The gold nanoparticles, at different stages of the coating process, were dimensionally and morphologically examined using a Hitachi High-Tech HT7700 microscope, in a 100 kV High Resolution Mode. Small amounts of aqueous samples (15 µL) were dropped on carbon coated grids (Ted Pella), being left at room temperature until complete evaporation of the solvent. The TEM images were further analyzed using imageJ software to assess the size distribution of the nanoparticles. The histograms indicating dimensional distribution of the nanoparticles were obtained by measurements made on about 1000 particles from several TEM images, of which only representative ones will be presented.

#### The hydrodynamic diameter and ζ-potential measurements

The size and ζ-potential of gold nanoparticles were investigated by dynamic light scattering (DLS) and electrophoretic light scattering (ELS), respectively, using the DelsaNano C Submicron Particle Size Analyzer (Beckman Coulter), equipped with dual 30 mW laser diodes emitting at 658 nm. The measurements were performed at 25 °C and neutral pH, and repeated three times for each sample. The colloidal suspensions were sonicated using a Branson 200 ultrasonic bath (Branson Ultrasonic Corp.) for 1 min.

### Electrochemical characterization

Cyclic voltammetry (CV) was used to evaluate the electrochemical behavior of gold nanoparticles (*AuNPs*, *AuPEG*_*2000*_*-NH*_*2*_ and *AuPEG*_*2000*_*-NH*_*2*_*-DOX*, irradiated compared with non-irradiated) deposited by physical adsorption (3 µL) from aqueous solution on the screen-printed electrode with a planar configuration, fabricated and procured from Biosensor Laboratory, University of Florence, Italy. The working electrode is of a disk shape of 3 mm diameter made of carbon paste, while the silver reference and carbon counter electrode were symmetrically placed around working electrode. The voltammetry assays were carried out using AUTOLAB PGSTAT302N instrument from ECO CHEMIE Utrecht, The Netherlands, and a single compartment electrochemical cell of 4 ml H_2_SO_4_ 0.05 M.

### Biological assay on culture cell

Cell cultures: HOS and NHDF cells were grown in tissue culture flasks with alpha-MEM medium supplemented with 10% FBS and 1% Penicillin-Streptomycin-Amphotericin B mixture. The medium was refreshed every 3 or 4 days. Once cells have reached confluency, they were detached with 1x Trypsin-Versene, washed with PBS, centrifuged at 200 x g for 3 minutes and subcultured into new tissue culture flasks.

The cytotoxicity of the samples was evaluated using the CellTiter 96® Aqueous One Solution Cell Proliferation Assay. HOS and NHDF cells were seeded into a 96-well culture plate at a density of 1 × 10^4^ cells per well and NHDF cells were seeded at a density of 5 × 10^3^ cells per well, in 100 µl culture medium (alpha-MEM medium supplemented with 10% FBS and 1% Penicillin-Streptomycin-Amphotericin B mixture). After 24 hours the medium was replaced with fresh media containing various concentrations of samples (10, 1, 0.5, 0.1, 0.05, 0.01 μg/mL), while the control group received only cell culture medium. The experiment was repeated three times, and for each sample minimum 3 replicates were accomplished. After 46 hours, 20 μL of CellTiter 96® Aqueous One Solution reagent was added to each well, and the plates were incubated for another 2 hours before reading the result. The absorbance was recorded at 490 nm using a plate reader (EnSight, PerkinElmer). Cell viability was calculated and expressed as percentage relative to viability of control cells (considered 100%).

## Supplementary information

Supplementary Information.

## Data Availability

All data generated or analyzed during this study are included in this published article and its Supplementary Information file.
