# Supplementary material for: Effect of TAT-DOX-PEG irradiated gold nanoparticles conjugates on human osteosarcoma cells
Source: Sci Rep. 2020 Apr 20;10:6591. doi: 10.1038/s41598-020-63245-8 (PMC7171153; doi:10.1038/s41598-020-63245-8)
Supplement: Supplementary file 1 — Supplementary Information. [file 41598_2020_63245_MOESM1_ESM.pdf]

SUPPLEMENTARY INFORMATION

Effect of TAT-DOX-PEG irradiated gold nanoparticles conjugates on human osteosarcoma cells

Raoul V. Lupusoru,[1,#] Daniela A. Pricop,[2,#] Cristina M. Uritu,[3,4,*] Adina Arvinte,[3] Adina Coroaba,[3,*] Irina Esanu,[5] Mirela F. Zaltariov,[6] Mihaela Silion,[3] Cipriana Stefanescu,[7] Mariana Pinteala,[3,*]

[1]Department of Pathophysiology, Faculty of Medicine, "Grigore T. Popa" University of Medicine and Pharmacy, 700115 Iasi, Romania

[2]Faculty of Physics, "Alexandru Ioan Cuza" University, 700506 Iasi, Romania

[3]Centre of Advanced Research in Bionanoconjugates and Biopolymers, "Petru Poni" Institute of Macromolecular Chemistry, 700487 Iasi, Romania

[4]Advanced Research and Development Center for Experimental Medicine (CEMEX), "Grigore T. Popa" University of Medicine and Pharmacy, 700115 Iasi, Romania

[5]Department of Internal Medicine I, "Grigore T. Popa" University of Medicine and Pharmacy, 700115 Iasi, Romania

[6]Department of Inorganic Polymers, "Petru Poni" Institute of Macromolecular Chemistry, 700487 Iasi, Romania

[7]Department of Biophysics and Medical Physics-Nuclear Medicine, "Grigore T. Popa" University of Medicine and Pharmacy, 700115 Iasi, Romania

[#]These authors contributed equally to this work.

***Corresponding authors*: MARIANA PINTEALA**, "Petru Poni" Institute of Macromolecular Chemistry, 41A Grigore Ghica Voda Alley, 700487 Iasi, Romania, e-mail: pinteala@icmpp.ro; **CRISTINA M. URITU**, "Grigore T. Popa" University of Medicine and Pharmacy, 16 Universitatii Street, 700115 Iasi, Romania, e-mail: cristina-mariana.uritu@umfiasi.ro; **ADINA COROABA**, "Petru Poni" Institute of Macromolecular Chemistry, 41A Grigore Ghica Voda Alley, 700487 Iasi, Romania, e-mail: adina.coroaba@icmpp.ro.

I. Supplementary schemes

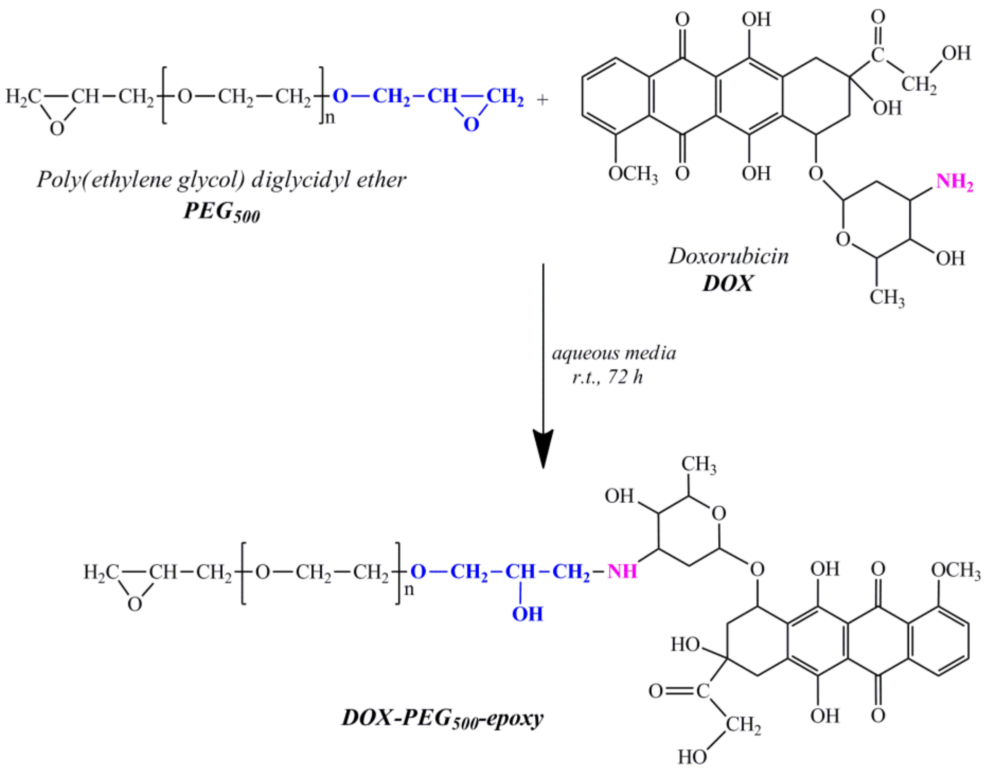

Scheme S1. The reaction scheme between DOX and PEG_{500} via oxirane opening ring, in aqueous media, at room temperature, for 72 h

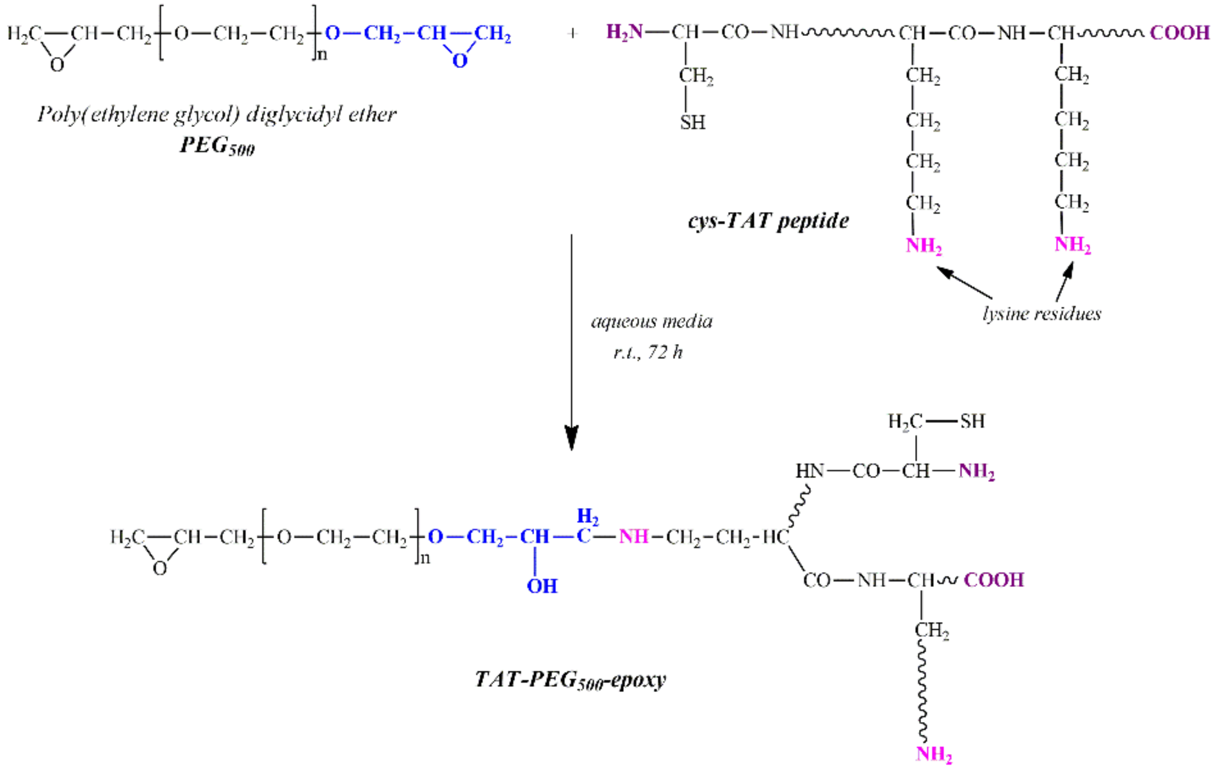

Scheme S2. The reaction scheme between cys-TAT and PEG_{500} via oxirane opening ring, in aqueous media, at room temperature, for 72 h

II. Ultraviolet-visible spectroscopy (UV-Vis)

Experimental

UV-Vis absorption spectra were recorded using a Shimadzu Spec Pharma 1800 instrument. The samples were 1/10 diluted to the initial concentration of the particle suspensions before recording the spectra. The resulting concentrations of the particle samples were calculated using Lambert-Beer law, based on the mean diameter provided by TEM imaging data[1].

Results and discussion

1. Structure characterization of AuNPs, *i*AUNPs, $AuPEG_{2000}$-NH_2 and *i*$AuPEG_{2000}$-NH_2 nanoparticles by UV-Vis spectroscopy

UV-Vis spectroscopy is well-known to assess the gold nanoparticle characteristics, principally related to size and concentration, in close connection with imaging data[2,3]. Exposing the samples in the wavelength range between 300 and 700 nm, the corresponding spectra were obtained, providing the absorption band of each compound as shown in Figure S1. One can observe that the intensity of the absorption band intensity (SPR) has increased in irradiated sample *iAuNPs* as compared to non-irradiated one, suggesting the continuation of nucleation process after light absorption[4]. The red shift in the SPR absorbance of the nanoparticles with polymer was largely determined by a slight change in the refractive index of the local environment of the AuNPs, indicating an increase in nanoparticle size[5]. At the same time, a slight increase in the SPR bandwidth was explained by the amplification of local plasmonic field, which may be due to the dimerization tendency of irradiated nanoparticles[6], as confirmed by TEM images. The UV-vis data shows that the SPR intensity of nanoparticles has changed after polymer coating. The red shift in the SPR absorbance of the PEGylated nanoparticles was largely determined by a slight change in the refractive index of the local environment of the *i*$AuPEG_{2000}$-NH_2, indicating an increase in nanoparticle size[5]. According to Figure S1, the spectrum of *i*$AuPEG_{2000}$-NH_2 suspension reveals a maximum intensity of the SPR band at 528 nm, while those of $AuPEG_{2000}$-NH_2 at 536 nm. The red shifted absorption bands of the PEGylated nanoparticle spectra are evidence regarding the successful grafting of the polymer onto the gold nanoparticle surface, either native or irradiated.

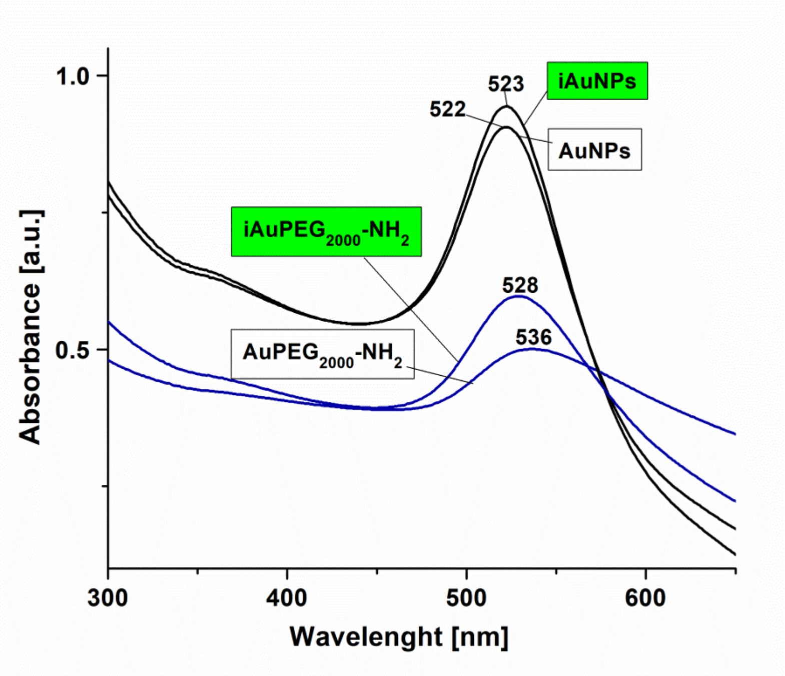

Figure S1. UV-Vis spectra of AuNPs, *i*AUNPs, AuPEG$_{2000}$-NH$_2$ and *i*AuPEG$_{2000}$-NH$_2$ nanoparticles displaying the influence of irradiation on spectral features. The spectrum of *i*AuPEG$_{2000}$-NH$_2$ is less shifted and shows an absorption band of higher intensity than in AuPEG$_{2000}$-NH$_2$.

2. Determination of the concentration of intermediate products, AuPEG$_{2000}$-NH$_2$ and *i*AuPEG$_{2000}$-NH$_2$using a calibration curve in UV-Vis.

After purification by centrifugation, the excess of unbound ligand and displaced citrate were removed along with a percentage of gold particles that should be quantified. The calibration curves were obtained for each product, by measuring the absorbance at 536 and 528 nm for AuPEG$_{2000}$-NH$_2$ and *i*AuPEG$_{2000}$-NH$_2$, having different, known concentrations (see Table S1 and S2).

The absorbance values of purified nanoparticles were found of 0.838 and 1.421 a.u. for AuPEG$_{2000}$-NH$_2$ and *i*AuPEG$_{2000}$-NH$_2$, respectively. Using the corresponding equations of the calibration curves (see Fig. S2 and S3), one can assess that the concentrations of the aforementioned products are around 0.2 mM in both cases, indicating a percentage of 80% recovered nanoparticles after polymer coating.

Table S1*. UV-Vis spectroscopy data obtained for different concentrations of AuPEG$_{2000}$-NH$_2$*

Concentration [mM]	0.02	0.04	0.08	0.1	**X**	0.25
Absorbance [a.u.]	0.108	0.187	0.367	0.407	**0.838**	1.0159

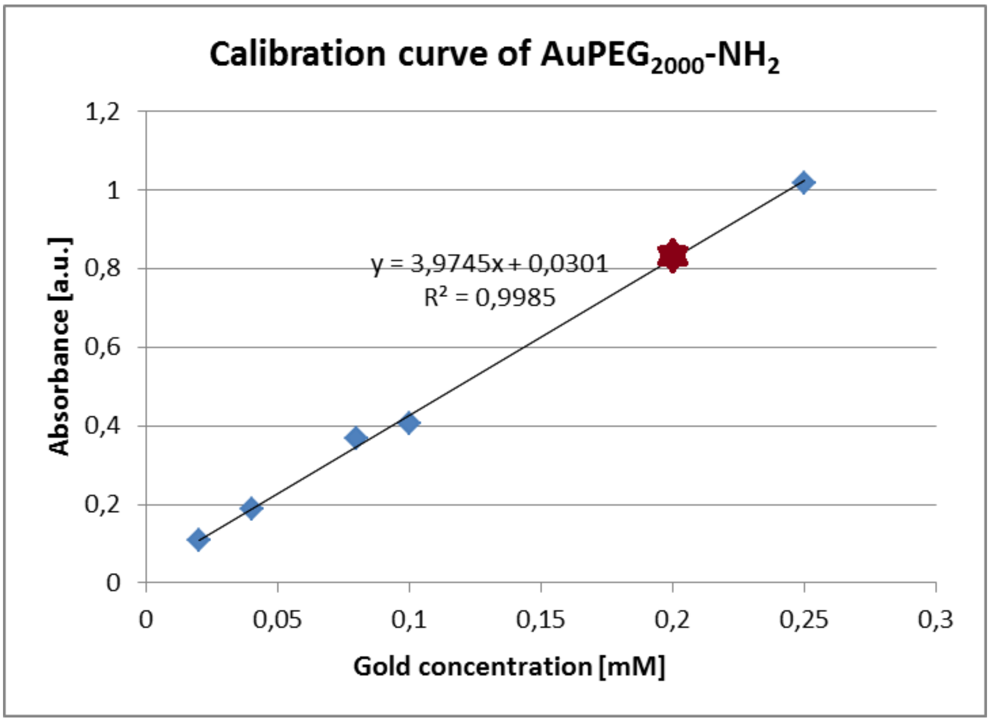

Figure S2. The absorbance calibration curve of *AuPEG*$_{2000}$*-NH*$_2$ based on the data from Table S1.

Table S2. *UV-Vis spectroscopy data obtained for different concentrations of iAuPEG*$_{2000}$*-NH*$_2$

Concentration [mM]	0.02	0.04	0.08	0.1	**X**	0.25
Absorbance [a.u]	0.186	0.293	0.59	0.748	**1.421**	1.765

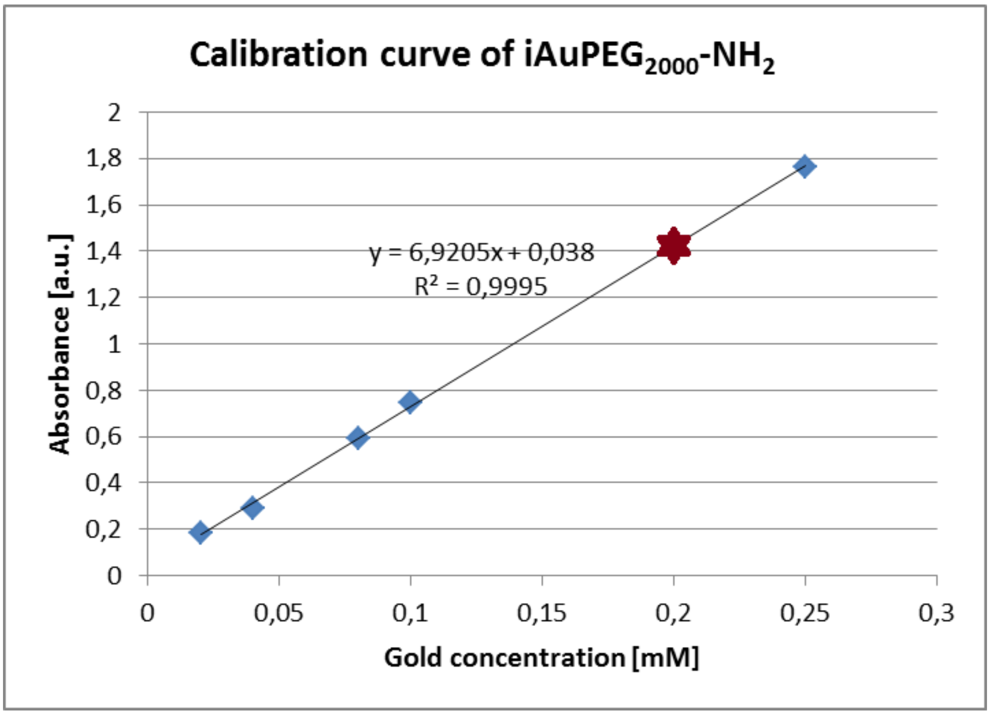

Figure S3. The absorbance calibration curve of *iAuPEG*$_{2000}$*-NH*$_2$ based on the data from Table 2.

III. Mass spectrometry (MS) assay

Experimental

MS data were acquired using an Agilent 6520 Series Accurate-Mass Quadrupole Time-of-Flight (Q-TOF) LC/MS instrument. The aqueous solutions of *DOX-PEG$_{500}$-epoxy* and *TAT-PEG$_{500}$-epoxy* precursors were studied by MS analysis along with the aqueous solutions of starting compounds (DOX, cys-TAT and PEG$_{500}$). The samples were injected into the electrospray ion source (ESI) using a syringe pump at a flow-rate of 0.01 mL·min^{-1}. The running parameters of Q-TOF MS were set as follows: electrospray ionization in positive ion mode; drying gas (N_2) flow rate 8 L·min^{-1}; drying gas temperature 325 °C; nebulizer pressure 25 psig; capillary voltage 4000 V; fragmentation voltage 200 V; the full-scan mass spectra of the examined compounds were acquired in the m/z range 100–3000. The mass scale was calibrated using the standard calibration procedure and standard compounds provided by the manufacturer. Data were collected and processed using Mass Hunter Workstation Software Data Acquisition for 6200/ 6500 Series, version B.01.03.

Results and discussion

PEG$_{500}$ precursor can be easily ionized under positive conditions and all peaks present in the mass spectrum (Fig. S4a) were assigned to single-charged sodium adduct ions series $[M+Na]^+$ with repeating units of 44 Da (C_2H_4O monomer unit).

The DOX drug (Fig. S4b) was detected at m/z 544.21, as single charge protonated ion $[DOX + H]^+$, corresponding to its molecular mass of 543 Da. Mass spectrum also shows the existence of a dimeric species $[2DOX + H]^+$ at m/z 1087.39. In addition to the signal corresponding to the DOX monomeric and dimeric species, the ion at m/z 397.11 occurs due to the loss of amino sugar moiety, corresponding to 146 Da. The mass spectrum of the DOX-PEG$_{500}$-epoxy (Fig. S4c) revealed the formation of complex at m/z 1026.57, corresponding to single charged protonated ion [DOX-PEG$_{500}$-epoxy+H]$^+$, which confirms that the reaction of DOX with PEG$_{500}$-epoxy was successfully carried out. However, in the mass spectrum of DOX-PEG$_{500}$-epoxy it can be also observed low-intensity ion peaks of unreacted precursors.

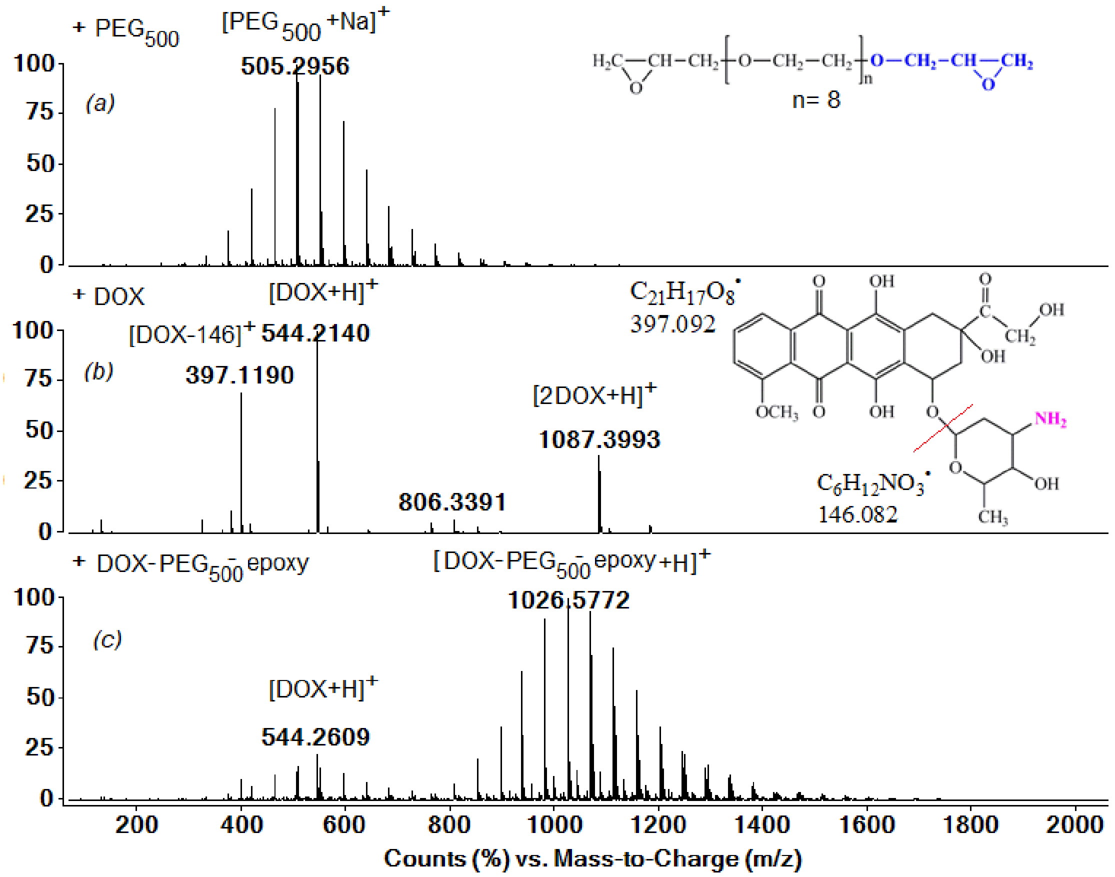

Figure S4. Positive ESI-QTOF mass spectra of (a) PEG_{500}, (b) DOX and (c) DOX-PEG_{500}-epoxy.

The TAT molecules show multiple charge states in a mass spectrum (Fig. S5a), where the distinctive ions at m/z 439.69, 658.51 and 1315.97 are due to the formation of the single, doubly and triply charged ions of TAT peptide, $[TAT+H]^{+}$, $[TAT+2H]^{2+}$ and $[TAT+3H]^{3+}$, respectively.

The ESI-MS spectrum of TAT-PEG_{500}-epoxy (Fig. S5b) shows the presence of the molecular ion of the complex at low intensity m/z 1820.17 corresponding to single-charge sodium adduct (these ions were underlined in the zoom). In addition, the peaks at m/z 329.5, 439.33, 658.51 and 1315.97 corresponds to unreacted TAT (multiple charge states up to +4).

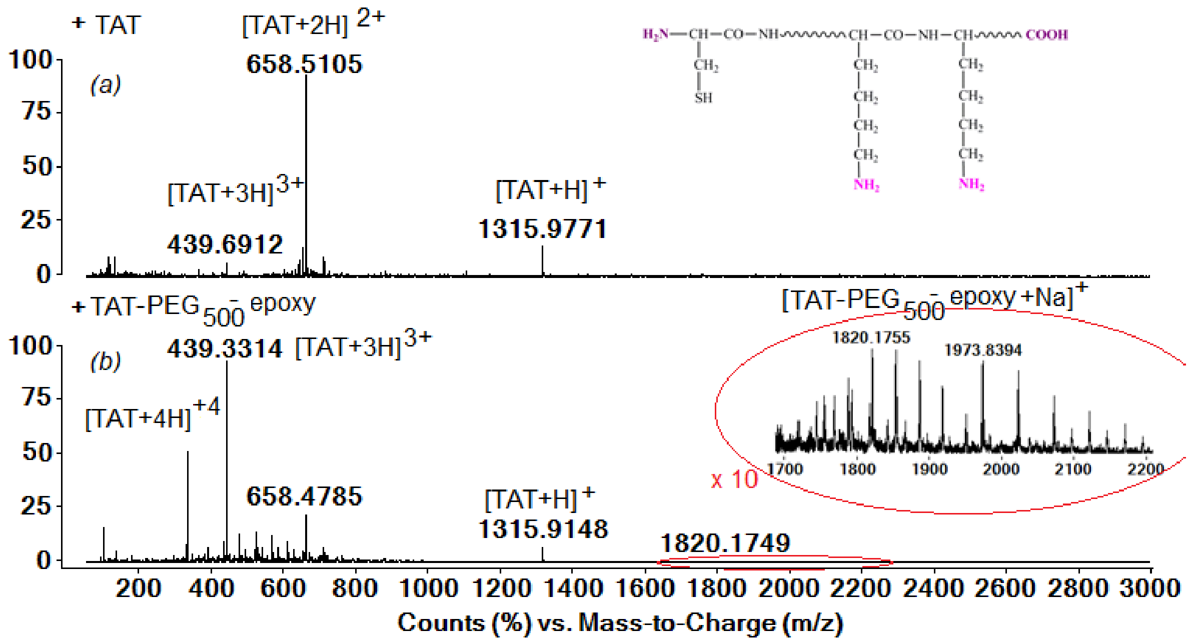

Figure S5. Positive ESI-QTOF mass spectra of (a) TAT and (b) TAT-PEG$_{500}$-epoxy.

IV. Particle size and morphology of gold nanoparticles using TEM imaging

Supplementary figures and notes

To determine the mean diameter and dimensional distribution of the synthesized nanoscale entities, a series of microscopic images were analysed, measuring about 1000 particles per product. Figure S6 illustrates TEM images of PEGylated nanoparticles (irradiated and non-irradiated), along with the starting products (AuNPs and *i*AuNPs**),** using a scale of 100 nm scale to observe the morphological details, while in the main manuscript there were presented images at a scale of 200 nm to observe the agglomeration tendency, when applicable.

The Table S3 correlates the particles size and morphology data obtained by different techniques: UV-Vis spectroscopy, TEM, DLS/ELS.

Table S3. *The influence of irradiation and polymer coating on suspension properties.*

**Sample**	**λ_{max}** [nm] *UV-Vis*	**Mean diameter** [nm] *TEM*	**Hydrodynamic diameter** [nm] *DLS*	**Polydispesity Index** *DLS*	**ζ potential** [mV] *ELS*
**AuNPs**	522	16.83	39.0±5.6	0.45	-40.35
***i*AuNPs**	522	16.00	19.5±2.8 (>99%)<br>138.8±27.3	0.76	-30.10
**AuPEG$_{2000}$-NH$_2$**	536	21.5	43.3±0.0 (>99%)<br>461.3±93.7	0.57	26.97
***i*AuPEG$_{2000}$-NH$_2$**	528	22.05	44.9±3.4 (>99%)<br>401.7±93.8	0.46	29.07

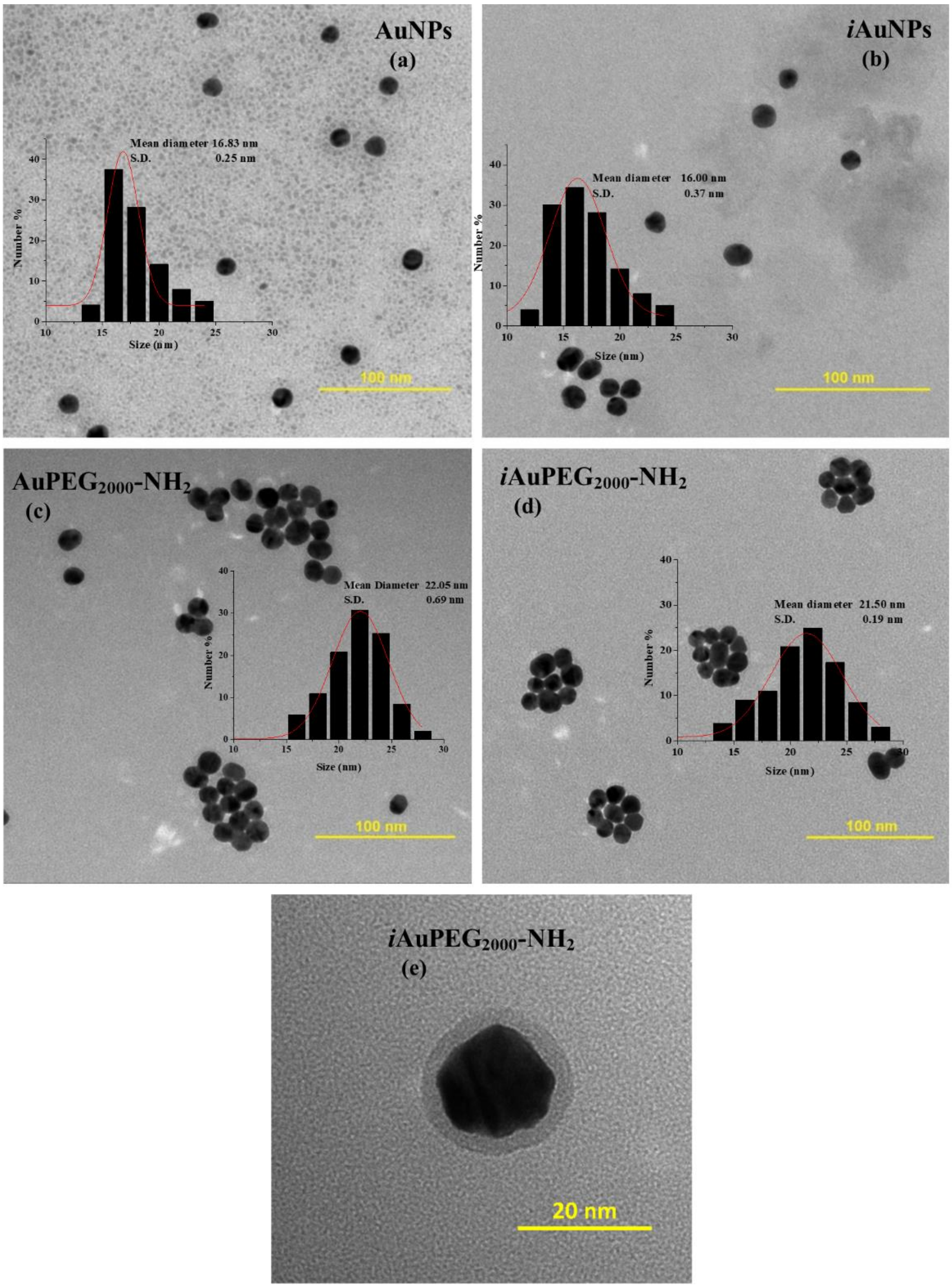

Figure S6. TEM images of non-irradiated particles, AuNPs (a) and $AuPEG_{2000}$-NH_2 (c), in comparison with irradiated products, *i*AuNPs (b) and *i*$AuPEG_{2000}$-NH_2 (d).
The polymer coating (e) has a slight influence on the particle size, but showing a more significant effect on aggregation behaviour. The irradiated PEGylated nanoparticles exhibit a phenomenon of uniform clustering, with the formation of entities with dimensions up to 100 nm.

V. Fourier-transform infrared spectroscopy (FTIR)

Supplementary results and discussions

The variation of FTIR spectra for the intermediate products AuNPs, *i*AuNPs, $AuPEG_{2000}$-NH_2 and *i*$AuPEG_{2000}$-NH_2 are presented in Figure S7, beside sodium citrate and HS-PEG_{2000}-NH_2.

Usually, the absorption of citrate molecules by specific coordination of carboxylate is dominant, but a number of other citrate molecules are subjected to intermolecular interactions with adsorbed species, which are not in contact with metal surface[7]. The mode of the carboxylate binding (bridging and chelating) was determined by the magnitude of separation between the carboxylate stretches. The FTIR spectrum of the pure sodium citrate shows two distinct absorption bands assigned to asymmetric and symmetric stretching vibrations of COO^- at 1616 and 1398 cm^{-1}, respectively. In AuNPs one can be observed two similar absorption bands, slightly shifted to 1591 and 1396 cm^{-1}, while in the IR spectrum of *i*AuNPs the asymmetric stretching vibration appeared at 1631 cm^{-1} and the symmetric stretches are present at 1371 and 1450 cm^{-1} revealing two coordination mode of the carboxylate binding: bridging and monodentate. The magnitude of separation between the carboxylate stretches are: $\Delta_{\text{sodium citrate}}$=218 cm^{-1} (ionic), Δ_{AuNPs}=195 cm^{-1} (bridging), Δ_{iAuNPs}=181 cm^{-1} (bridging) and monodentate ($\Delta_{i\text{AuNPs}}$=260 cm^{-1}), following the generally proposed order: Δ(chelating) < Δ(bridging) < Δ(ionic) < Δ(monodentate)[8]. A weak peak at 1734-1735 cm^{-1} can be observed in all nanoparticle compounds and was assigned to C=O stretching vibrations of carboxyl group. The PEGylated products do not exhibit significant differences between $AuPEG_{2000}$-NH_2 and *i*$AuPEG_{2000}$-NH_2, but the FTIR signal of PEG has undergone several modifications. The C-O-C stretching bands of HS-PEG_{2000}-NH_2 were found at 1280 and 1112 cm^{-1}, while in PEGylated gold nanoparticles one can be observed only a strong peak at 1151 and 1149 respectively, with a shoulder at 1130 cm^{-1}. The aliphatic C-H bending vibrations, encountered at 1400 and 1344 cm^{-1} in HS-PEG_{2000}-NH_2 as moderate intensity peaks, were found at 1400 cm^{-1} as strong peaks in conjugated compounds. The C-H stretching vibrations at 2887 cm^{-1} as strong signal in unreacted HS-PEG_{2000}-NH_2 are smaller and a little shifted at 2852, 2924 and 3014 cm^{-1} in $AuPEG_{2000}$-NH_2 and at 2854, 2924 and 3016 cm^{-1} in *i*$AuPEG_{2000}$-NH_2. C-H rocking vibrations observed at 1469 cm^{-1} in HS-PEG_{2000}-NH_2 cannot be distinguished in the products. The primary amino group is very clearly represented in the spectra of all PEGylated compounds by the characteristic bands at 3126 cm^{-1} and 1629 cm^{-1}. In the spectra of non-irradiated and irradiated $AuPEG_{2000}$-NH_2 conjugates the well-defined band at 696 nm is due to the formation of Au-S bonds and the smaller one at 796 nm is attributed to the C-S bond. The Table S4 summarizes all FTIR data obtained and peak assignment.

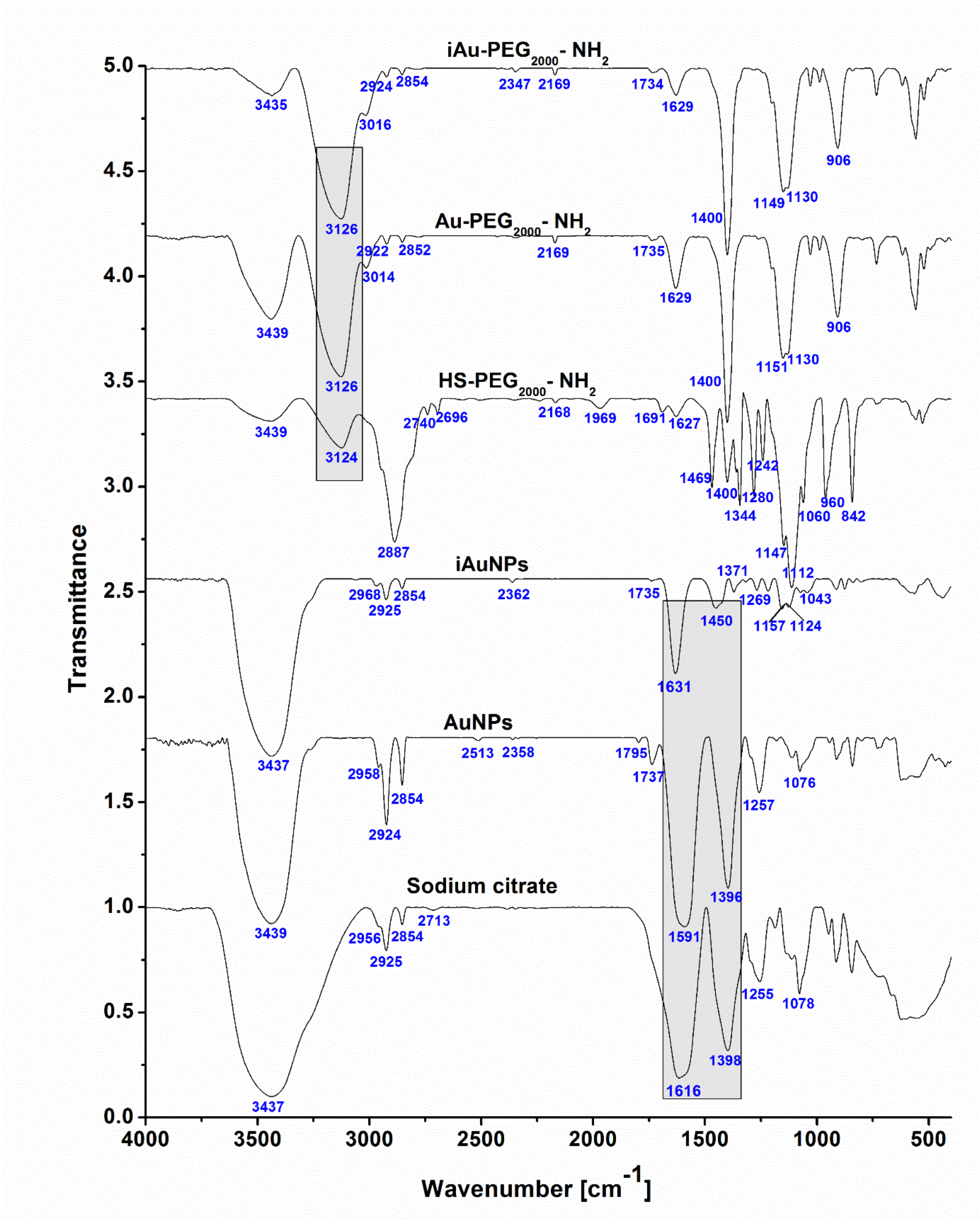

Figure S7. FTIR spectra of AuNPs, *i*AuNPs, AuPEG$_{2000}$-NH$_2$ and *i*AuPEG$_{2000}$-NH$_2$ samples besides sodium citrate and PEG$_{2000}$-NH$_2$

Table S4. *FTIR spectra band assignment for non-irradiated and irradiated samples.*

Band assignment	Band (cm^{-1})						
		Non-irradiated samples			Irradiated samples		
	**DOX-PEG$_{500}$-epoxy**	**AuPEG$_{2000}$-NH$_2$**	**AuPEG$_{2000}$-DOX**	**AuPEG$_{2000}$-TAT-DOX**	***i*AuPEG$_{2000}$-NH$_2$**	***i*AuPEG$_{2000}$-DOX**	***i*AuPEG$_{2000}$-TAT-DOX**
**CH_2 rocking**		426		433	428		433
**C-C stretching**	464			464		462	466
**C-S stretching**		489	478		493		
**C-C vibrations**	518	520			520		536
			543	540		540	
		557			557		
			594	584			
**C-H**	617	619	621	619	617	622	615
**Au-S**		696	688	696	698	698	690
**N-H bending**	725	732			732		
**C-S, N-H**		796	779		798		785
	804			796		800	
**C-H rocking**			839	840	839	842	842
**C-O stretching**		906			906		
**C-OH, C-O-C deformation**	948		950	948		950	950
		987			987		
**-C-O stretching**	1031	1028			1029	1031	
**C-O-C, C-C-H stretching**	1109		1107	1109		1101	1109
		1130			1130		
		1151			1149		
**C-O, C-N**	1207	1203		1203	1203	1211	1203
	1259		1259	1257		1259	1257
**C-H bending, amide III**	1402	1400	1390	1392	1400	1396	1392
**C-H bending**	1452		1458	1458		1462	1458
**C=O, amide II**	1585		1577	1583		1575	1587
**N-H deformation, amide I**	1620	1627	1625		1629	1639	
				1674			1668
**C=O stretching**	1726	1736	1735	1739	1733	1735	1735
**C-H stretching**	2862	2852	2862	2866	2854	2856	2862
	2924	2924	2925	2924	2924	2924	2924
		3014			3016		
**N-H primary**		3126			3126		
				3207			3210
**N-H stretching**	3246						
				3321			3307
	3386			3375			
							3398
**-OH**		3438	3425		3435	3467	

VI. XPS spectroscopy

Table S5. *The assignments of XPS signals from deconvoluted C1s and Au4f high resolution spectra of AuNPs, iAUNPs, AuPEG2000-NH2 and iAuPEG2000-NH2 products.*

Sample	High resolution spectrum	Binding energy (eV)	Assign.	Area %	High resolution spectrum	Binding energy (eV)	Assign.	Area %
**AuNPs**	C1s	284.6<br>286.0<br>287.9<br>289.2	C-H/C-C<br>C-O<br>COO^-<br>COOH	66.93<br>11.81<br>17.71<br>03.53	Au4f	82.7<br>86.4<br>83.4<br>87.1	Au^0<br><br>Au^+	95.50<br><br>4.50
***i*AuNPs**	C1s	284.6<br>286.1<br>287.7<br>288.9	C-H/C-C<br>C-O<br>COO^-<br>COOH	63.03<br>15.68<br>15.96<br>05.34	Au4f	82.7<br>86.4<br>83.6<br>87.3	Au^0<br><br>Au^+	80.45<br><br>19.55
**$AuPEG_{2000}$-NH_2**	C1s	284.6<br>286.2<br>287.4<br>288.2<br>289.0<br>289.5	C-C/C-H<br>C-O<br>COO^-<br>C-N<br>C-S<br>COOH	54.37<br>16.21<br>04.32<br>06.03<br>06.03<br>13.05	Au4f	82.9<br>86.6<br>83.4<br>87.1<br>83.7<br>87.4	Au^0<br><br>Au^+<br><br>Au-S	87.66<br><br>4.62<br><br>7.72
***i*$AuPEG_{2000}$-NH_2**	C1s	284.6<br>286.1<br>287.4<br>288.2<br>289.0<br>289.6	C-C/C-H<br>C-O<br>COO^-<br>C-N<br>C-S<br>COOH	65.66<br>09.30<br>02.63<br>06.73<br>06.73<br>08.96	Au4f	82.9<br>86.6<br>83.2<br>86.9<br>83.5<br>87.2	Au^0<br><br>Au^+<br><br>Au-S	78.15<br><br>15.63<br><br>6.23

VII. Biological assay

Preparation of solutions for the MTS assay. Determination of nanoparticle loading with drug.

Firstly, the mass concentration of the final products has been determined. The total amount of *$AuPEG_{2000}$-TAT-DOX* in a volume of 1058.33 µL solution (1000 µL *$AuPEG_{2000}$/i$AuPEG_{2000}$*, 50 µL *DOX-PEG_{500}-epoxy* and 8.33 µL *TAT-PEG_{500}-epoxy*), identical to that of *i$AuPEG_{2000}$-TAT-DOX* is 8.6029 mg, calculated by summing the weight of all components present in the reaction mixture: 0.0394 mg Au ($0.2\cdot10^{-3}$ mmol · 197 g/mol), 8 mg PEG_{2000} ($4\cdot10^{-3}$ mmol · 2000 g/mol), 0.48 mg *DOX-PEG_{500}-epoxy* ($0.46\cdot10^{-3}$ mmol · 1043.5 g/mol, M_{DOX} = 543.5 g/mol) and 0.0835 mg *TAT-PEG_{500}-epoxy* ($0.046\cdot10^{-3}$ mmol · 1815 g/mol, $M_{cys\text{-}TAT}$ = 1315 g/mol). Thus, the concentration of these solutions was calculated, having the value of 8.13 mg/mL. Two stock solutions (1000 µL) of 1 mg/mL *$AuPEG_{2000}$-TAT-DOX/ i$AuPEG_{2000}$-TAT-DOX* were prepared by mixing 123 µL of the 8.13 mg/mL solutions with 877 µL ultrapure water for each of them. Similarly, the total amount of *$AuPEG_{2000}$-DOX* in a volume of 1050 µL solution, identical to that of *i$AuPEG_{2000}$-DOX* is 8.5165 mg, calculated by summing the all the components present in the reaction mixture: 0.0394 mg Au

($0.2\cdot10^{-3}$ mmol · 197 g/mol), 8 mg PEG_{2000} ($4\cdot10^{-3}$ mmol · 2000 g/mol) and 0.48 mg *DOX-PEG₅₀₀-epoxy* ($0.46\cdot10^{-3}$ mmol · 1043.5 g/mol, M_{DOX} = 543.5 g/mol). The corresponding concentration of these solutions was found of 8.11 mg/mL. Another two stock solutions (1000 µL) of 1 mg/mL *$AuPEG_{2000}$-DOX*/ *i$AuPEG_{2000}$-DOX* have been prepared mixing 123.31 µL of the 8.11 mg/mL solutions and 876.69 µL ultrapure water for each of them. Solutions of concentrations of 100, 10, 1 and 0.01 µg/mL were further prepared by successive dilutions of the stock solutions (1 mg/mL) for all compounds in question. Since the antitumor activity of drug loaded nanoparticles was evaluated against the pure drug having the same concentration, the percent of the drug from the carrier was also calculated. Thus, the *$AuPEG_{2000}$-TAT-DOX*/*i$AuPEG_{2000}$-TAT-DOX* compounds, comprising 0.25 mg DOX ($0.46\cdot10^{-3}$ mmol · 543.5 g/mol) from the total amount of product (8.6029 mg), which is equivalent to 2.9% of the drug covalently bound in the nanoparticulate coating. For those products containing no TAT peptide, mL *$AuPEG_{2000}$-DOX* and *i$AuPEG_{2000}$-DOX*, this percentage is very slightly higher, 2.94% respectively (0.25 mg DOX from 8.5165 mg product). As a consequence, the solutions of 10, 1 and 0.01 µg/mL drug loaded carrier comprise 0.29/0.294, 0.029/0.0294 and 0.0029/0.00294 µg/mL DOX, respectively.

References

1. Liu, X., Atwater, M., Wang, J. & Huo, Q. Extinction coefficient of gold nanoparticles with different sizes and different capping ligands. *Colloids Surf. B Biointerfaces* **58**, 3–7 (2007).
2. Aslam, M., Fu, L., Su, M., Vijayamohanan, K. & Dravid, V. P. Novel one-step synthesis of amine-stabilized aqueous colloidal gold nanoparticles. *J. Mater. Chem.* **14**, 1795–1797 (2004).
3. Ji, X. *et al.* Size control of gold nanocrystals in citrate reduction: the third role of citrate. *J. Am. Chem. Soc.* **129**, 13939–13948 (2007).
4. Andries, M., Pricop, D., Oprica, L., Creanga, D.-E. & Iacomi, F. The effect of visible light on gold nanoparticles and some bioeffects on environmental fungi. *Int. J. Pharm.* **505**, 255–261 (2016).
5. Venkatesan, R. *et al.* Doxorubicin conjugated gold nanorods: a sustained drug delivery carrier for improved anticancer therapy. *J. Mater. Chem. B* **1**, 1010–1018 (2013).
6. Boerigter, C., Campana, R., Morabito, M. & Linic, S. Evidence and implications of direct charge excitation as the dominant mechanism in plasmon-mediated photocatalysis. *Nat. Commun.* **7**, 10545 (2016).
7. Martynyuk, O. *et al.* On the high sensitivity of the electronic states of 1 nm gold particles to pretreatments and modifiers. *Molecules.* **21**, 432 (2016).

8. Zeleňák, V., Vargová, Z. & Györyová, K. Correlation of infrared spectra of zinc(II) carboxylates with their structures. *Spectrochim. Acta A Mol. Biomol. Spectrosc.* **66**, 262–272 (2007).
